# Adult asthma and traffic exposure at residential address, workplace address, and self-reported daily time outdoor in traffic: A two-stage case-control study

**DOI:** 10.1186/1471-2458-10-716

**Published:** 2010-11-22

**Authors:** Anna Lindgren, Jonas Björk, Emilie Stroh, Kristina Jakobsson

**Affiliations:** 1 Department of Occupational and Environmental Medicine, Lund University, Sweden

## Abstract

**Background:**

Most epidemiologic studies use traffic at residential address as a surrogate for total traffic exposure when investigating effects of traffic on respiratory health. This study used GIS (Geographical Information Systems) to estimate traffic exposure, not only on residential, but also on workplace address, in addition to survey questions on time spent in traffic during commuting or other daily activities.

The aim was to investigate 1) if there is an association between traffic exposure and prevalence of adult asthma and asthma symptoms, and 2) if so, does this association become stronger using more complete traffic exposure information.

**Methods:**

This study was conducted in two stages: A first cross-sectional survey in Southern Sweden 2004 (n = 24819, 18-80 years, response rate 59%) was followed by a case-control study in 2005 to obtain more detailed exposure and confounder information (n = 2856, asthmatics and controls (1:3), 86% response rate). In the first survey, only residential address was known. In the second survey, questions about workplace addresses and daily time spent in traffic were also included. Residential and workplace addresses were geocoded and linked with GIS to road data and dispersion modelled outdoor concentrations of NO_x _(annual mean, 250 × 250 m resolution).

**Results:**

Living within 50 m of a road (measured by GIS) with traffic intensity of >10 cars/minute (compared with no road within this distance) was associated with an increased prevalence of asthma, (OR = 1.8, 95% CI = (1.1-2.8), and with asthma symptoms last 12 months. No statistically significant effects were seen for traffic exposure at workplace address, daily time spent in traffic, or commuting time to work, after adjustment for confounders. A combined total exposure estimate did not give a stronger association with asthma prevalence or asthma symptoms.

**Conclusions:**

Traffic exposure at close proximity to residential address showed association with asthma prevalence and asthma symptoms last 12 months, among adults in southern Sweden. The associations were not stronger when accounting for total traffic exposure. This could reflect exposure misclassfication at workplace address and for other daily time in traffic, but also that residential address remains the main determinant for traffic exposure among adults.

## Background

That air pollution can trigger asthma symptoms is well known [1], and there is increasing evidence that traffic also induces asthma incidence in both children [2] and adults [3-6]. This increasing evidence from epidemiological studies has been parallel with and probably dependent on the development of long-term exposure measures of traffic with a geographically high spatial resolution, which capture contrasts in exposure better than data from air pollution monitor stations only [7].

Although the exposure models for traffic have becomes better in recent years, most studies still estimate only exposure to traffic at residential address, even if a large proportion of traffic exposure, especially for adults, is commuting time, and workplace exposure [8]. The misclassification from using residential exposure as a proxy for total exposure can be expected to distort the true risk estimates, and reduce the power to detect an effect [9]. While personal sampling exposure studies can estimate the relationship between traffic and respiratory symptoms in short-term studies, this is expensive and not feasible for longer time periods or larger populations. It can also be a disadvantage to measure concentrations of a specific pollutant from all sources, rather than the effects of a specific exposure source (i.e traffic) with its complex mixture. It has been suggested that geographical informations systems (GIS) should be used for dynamic, 24 h- modelling of long-term exposure from traffic [10], and this has been done in simulation studies [11], but empirical epidemiological studies linking this to health effects have been rare [12,13].

This is to our knowledge the first study on asthma and traffic to use GIS to estimate traffic exposure, not only at residential address, but also on workplace address and with self-reported information on commuting time to work or other outdoor activity in traffic. Traffic intensity and modelled outdoor NO_x _was used as proxies for local traffic-related air pollution, rather than exposure to NO_x _per se (which also comes from indoor sources like gas stoves). The aim was to investigate the association between traffic exposure and prevalence of asthma and asthma symptoms in adults in occupationally active age. We investigated 1) separate associations with traffic at residence, workplace, and daily time in traffic, and 2) if combining the exposures, i.e. accounting for total exposure, would strengthen the association between traffic and asthma.

## Methods

### Study area &sampling

**Figure 1:**
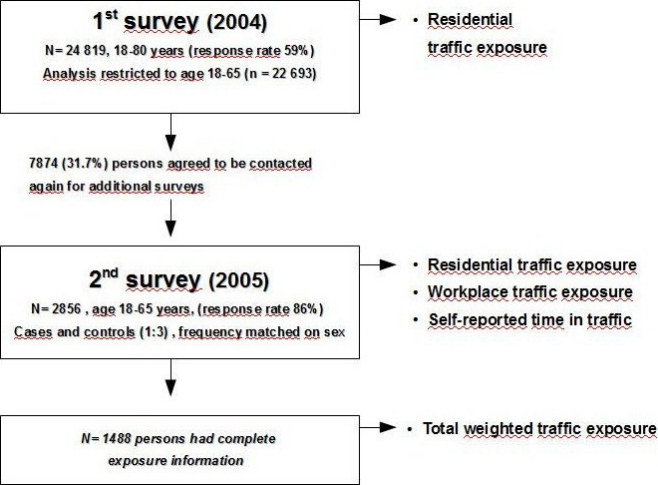
**Flow-chart of study design**.

This study was conducted in two stages (figure 1): A first large sample study was followed by nested sampling of a subgroup of asthma cases and controls for more detailed exposure assessment and confounder information.

The first study was a cross-sectional public health survey (Additional files 1, 2) conducted in Scania (southern Sweden) in 2004 (N = 24 819; 59% participation rate, age 18-80 years, however, we restricted our analysis to age 18-65 (n = 22693). The sampling was stratified by age, sex and geographical area, with equal number of subjects randomly sampled in each strata, independent on population size in order to increase the statistical power in some smaller administrative areas [14]. Thus, the descriptive data in the study are only representative for the entire Scania region in a weighted analysis. The survey had a broad public health purpose.

The sampling frame for the second survey (Additional files 3, 4) was those in the public health survey who had agreed to participate in additional studies (7874 persons, 31.7% of the participants in the first survey) and were in occupationally active age (18-65). The second survey was sent in 2005 to all eligible asthmatics and to controls (1:3, frequency matched on sex). The final case-control study included 2856 respondents (86% response rate), 705 asthmatics and the rest controls. The questions in this survey were focused on traffic exposures, housing conditions and occupational factors including information on workplace address.

The study was conducted in accordance with the Helsinki Declaration. No animals were used in the study and human subjects participated only after informed consent. Ethical permission for the study was obtained from the Regional Ethics Review Boards, Lund, Sweden. Reference number: dnr 387/2004.

### Geocoding

In the first survey, residential addresses for all participants were geocoded. For those participating in the second survey, workplace addresses were also geocoded. At residential address, geocoding was achieved by linking each individual's unique 10-digit personal identity codes to a registry containing the geographical coordinates of nationally registered residential address. This assigned individuals a position in the centre of their real estate.

Workplace addresses were obtained by self-report in the survey, and individuals were manually geocoded to this address, which is more accurate positioning than applying the centre of the real estate.

### Exposure assessment

All geocoded addresses were linked to GIS-based registers from the Swedish National Road Database, containing information about traffic intensity on all major roads in the county, for the year 2004. To assess exposure to traffic, we identified the road with the heaviest traffic intensity within 100 m of the residence. Traffic intensity was categorized as 0-1 cars/min, 2-5 cars/min, 6-10 cars/min, and >10 cars/min, based upon 24-hour mean levels.

All geocoded addresses were also linked to modelled concentrations of NO_x _based on a validated emission database based on year 2001 [15,16]. The exposure information for NO_x _is thus extrapolated from 2001. Emission sources included were: road traffic, shipping, aviation, railroad, industries and larger energy and heat producers, small scale heating, working machines, working vehicles and working tools. Meterological data were also included. A dispersion model (AERMOD) was used for dispersion calculation of annual mean concentrations μg/m^3^, within a 250 × 250 m grid, using bilinear interpolation. A detailed description and discussion of exposure assessment methods has been published previously [17].

In addition to GIS-estimated exposure, questions about traffic at residential address, traffic at workplace address and time spent in traffic were present in the second survey.

In total, the following exposures were investigated:

• *Residential address*.

GIS measured traffic intensity on the heaviest road within 50, 100, 250 m

GIS-modelled exposure to NO_x_

Survey question: "What is the traffic intensity on the heaviest road you can see from any window in your apartment? (within a distance of 50 m)"

• *Workplace address*.

GIS measured traffic intensity on the heaviest road within 50, 100, 250 m

GIS-modelled exposure to NO_x_

Survey question:" What is the traffic intensity on the street outside your work/school? (within a distance of 50 m)"

• *Daily activities*

Survey questions: "How much time do you on average spend outdoor in traffic every day? (in cars, buses, bike, walking on streets etc)?" and "How long time does it take for you to transport to work/school?"

• Total exposure. N = 1488 people had complete exposure information (geocoded residential and workplace address, reported time spent in traffic and reported percentage of full time work) and were thus used for calculation of total exposure.

Total exposure.was calculated as ((Total time - time at work - time in traffic)*NO_x _at home address) + (time at work*NO_x _at workplace address) + (time in traffic*C). The constant C representing the hypothesized average NO_x_-dose from time in traffic was varied between 30 and 300, since concentrations of fresh exhaust emissions like NO can be many times higher in curbside intense traffic, compared with background levels [18]. NO_x _at residential and workplace addresses were estimated by the GIS-modelling. Time in traffic was estimated from the survey question "How much time do you on average spend outdoor in traffic every day?". Time at work was estimated by reported percentage of full-time (40 h/week) occupation.

Categorisations of variables were chosen to be comparable with previous study in the area [17] and for the GIS-measures to be comparable with the self-reported questions. Information on years of living at current address was available.

### Outcome measures

The following questions were investigated, as obtained from the postal questionnaires:

• *Asthma prevalence*. "Do you have asthma?" The potential answers "No" "Yes, but no symptoms" "Yes, minor symptoms" "Yes, severe symptoms" were dichotomized to "No" and "Yes " (all three "Yes"-answers were categorized as "Yes"). This question was used in the first survey.

• *Asthma Symptoms during the last 12 months*. Have you had asthma symptoms during the last 12 months, i.e. intermittent breathlessness or attacks of breathlessness? This question was only used in the second survey.

Information about doctor's diagnosis of asthma and use of asthma medication was also available in the second survey.

### Statistical analyses

Univariate analyses of the association of asthma with the different traffic measures were performed. Analyses were also made restricted to those with asthma diagnosis, those with severe and minor symptoms, those with asthma medication (dichotomized as "no" versus "yes", where yes included both "yes, when needed" and "yes, regularly") and those which had been living >5 years at current address.

Associations between asthma and total exposure to NO_x _were also estimated. Traffic exposure was categorised into quantiles and effect estimates from total exposure was compared with effect estimates from quantiles based on the single-variate exposures. It could then be assessed if the association got stronger by reclassification of the same individuals according to complete exposure information. Odds Ratios (ORs) with 95% Confidence Intervals (CI) were estimated by binary logistic regression, using version 17.0 of SPSS.

Confounders which were known risk factors and present in both first and second survey were adjusted for (table 1). Adjusting for Socio-Economic Index (SEI) based on occupational status [19] and Body Mass Index (BMI) increased the effect estimates, while additional adjustment for the other confounders in table 1 did not change the estimates noticeably (below 10%), but these were still included in the model. Potential confounder variables from the second survey (damp, smell of mould, condensate on inside of window, more detailed work-exposure assessment by self-reported exposure to dust, motor exhaust or chemicals as separate entities, or by coding self-reported occupation to the ALOHA Job-Exposure-Matrix (JEM), showing probabilistic exposure to dust, gases or fumes [20]), did not noticeably change the estimate further and were not adjusted for.

**Table 1:** Descriptive data from the 1st and 2nd survey

		The 1^st ^survey (2004)			Non-cases stratified on exposure		The 2^nd ^survey (2005)			Non-cases stratified on exposure	
		Cases	Non-cases				Cases	Non-cases			
		n (%)	n (%)	OR	NOx <19	NOx >19	n (%)	n (%)	OR	NOx <19 μ/m^3^	NOx <19 μ/m^3^
**Sex**	Male	865 (40.4)	8726 (45.4)	1.0	7876 (45.5)	850 (44.9)	272 (39.0)	843 (39.0)	1.0	764 (39.3)	79 (36.6)
	Female	1275 (59.6)	10494 (54.6)	1.2 (1.1-1.3)	9449 (54.5)	1045 (55.1)	426 (61.0)	1317 (61.0)	1.0 (0.84-1.2)	1180 (60.7)	137 (63.4)
											
**Age **	18-24	282 (13.2)	2119 (11.0)	1.0	1867(10.8)	252 (13.3)	69 (9.9)	216 (10.0)	1.0	186 (9.6)	30 (13.9)
**(5 Groups)**	25-34	454 (21.2)	3521 (18.3)	0.97 (0.83-1.1)	2937 (17.0)	584 (30.8)	142 (20.3)	435 (20.1)	1.0 (0.73-1.4)	364 (18.7)	71 (32.9)
	35-44	395 (18.5)	4341 (22.6)	0.68 (0.58-0.80)	3980 (23.0)	361 (19.1)	139 (19.9)	470 (21.8)	0.93 (0.67-1.3)	436 (22.4)	34 (15.7)
	45-54	460 (21.5)	4276 (22.2)	0.81 (0.69-0.95)	3937 (22.7)	339 (17.9)	154 (22.1)	461 (21.3)	1.0 (0.75-1.5)	419 (21.6)	42 (19.4)
	55-65	549 (25.7)	4963 (25.8)	0.83 (0.71-0.97)	4604 (26.6)	359 (18.9)	194 (27.8)	578 (26.8)	1.1 (0.77-1.4)	539 (27.7)	39 (18.1)
											
**Smoking**	No	1630 (76.6)	14890 (77.9)	1.0	13556 (78.6)	1334 (70.7)	530 (76.3)	1690 (78.6)	1.0	1534 (79.2)	156 (72.6)
	Yes, sometimes	131 (6.2)	984 (5.1)	1.0 (0.92-1.2)	836 (4.8)	148 (7.8)	34 (4.9)	99 (4.6)	1.2(0.92-1.4)	84 (4.3)	15 (7.0)
	Daily	367 (17.2)	3250 (17.0)	1.2 (1.0-1.5)	2846 (16.5)	404 (21.4)	131 (18.8)	362 (16.8)	1.1(0.73-1.6)	318 (16.4)	44 (20.5)
											
**BMI**	< 25	1001 (48.4)	10325 (55.0)	1.0	9228 (54.5)	1097 (59.3)	315 (46.3)	1193 (56.2)	1.0	1066 (55.7)	127 (60.8)
	Overweight	740 (35.8)	6399 (34.1)	1.2 (1.1-1.3)	5821 (34.4)	578 (31.2)	264 (38.8)	676 (31.9)	1.5 (1.2-1.8)	614 (32.1)	62 (29.7)
	Fat	327 (15.8)	2057 (11.0)	1.6 (1.4-1.9)	1881 (11.1)	176 (9.5)	102 (15.0)	253 (11.9)	1.5 (1.2-2.0)	233 (12.2)	20 (9.6)
											
**SEI**	Professionals, etc	234 (11.8)	2333(12.9)	1.0	2132 (13.1)	201 (11.3)	81 (12.6)	307(14.9)	1.0	278 (15.0)	29 (14.3)
	Intermediate non-manual	340 (17.1)	3366 (18.7)	1.0 (0.85-1.2)	3086 (19.0)	280 (15.8)	124 (19.3)	436(21.2)	1.1(0.79-1.5)	399 (21.5)	37 (18.2)
	Assistant non-manual	187 (9.4)	1735 (9.6)	1.1 (0.88-1.3)	1559 (9.6)	176 (9.9)	71 (11.0)	188(9.1)	1.4 (0.99-2.1)	175 (9.4)	13 (6.4)
	Skilled workers	245 (12.3)	2359 (13.1)	1.0 (0.86-1.3)	2171 (13.3)	188 (10.6)	72 (11.2)	251(12.2)	1.1(0.76-1.6)	226 (12.2)	25 (12.3)
	Unskilled workers	334 (16.8)	3150 (17.5)	1.1 (0.89-1.3)	2854 (17.5)	296 (16.7)	100 (15.5)	287(13.9)	1.3 (0.95-1.8)	252 (13.6)	35 (17.2)
	Self-employed (non-prof.)	107 (5.4)	1275 (7.1)	0.8 (0.66-1.1)	1177 (7.2)	98 (5.5)	38 (5.9)	155(7.5)	0.93 (0.60-1.4)	145 (7.8)	10 (4.9)
	Disability pensioners	192 (9.7)	1042 (5.8)	1.8 (1.5-2.3)	927 (5.7)	115 (6.5)	66(10.2)	131(6.4)	1.9(1.3-2.8)	117 (6.3)	14 (6.9)
	Unemployed	139 (7.0)	1073 (5.9)	1.3 (1.0-1.6)	893 (5.5)	180 (10.2)	32(5.0)	107(5.2)	1.1 (0.71-1.8)	91 (4.9)	16 (7.9)
	Students	210 (10.6)	1702 (9.4)	1.2 (1.0-1.5)	1464 (9.0)	238 (13.4)	60(9.3)	197(9.6)	1.2(0.79-1.7)	173 (9.3)	24 (11.8)
Exposure to											
chemicals, dust, or fumes at work	Never	909 (61.7)	8876 (62.7)	1.0	8054 (62.4)	91 (62.3)	333 (65.7)	1085 (66.9)	1.0	988 (67.1)	97 (65.1)
	More seldom	303 (20.6)	2731 (19.3)	1.0 (0.86-1.2)	2511 (19.5)	30 (20.5)	101 (19.9)	282 (17.4)	0.97 (0.68-1.4)	256 (17.4)	26 (17.4)
	Few days/week	94 (6.4)	946 (6.7)	0.97 (0.78.-1.2)	872 (6.8)	8 (5.5)	28 (5.5)	103 (6.4)	0.89 (0.57-1.4)	91 (6.2)	12 (8.1)
	Every day	168 (11.4)	1597 (11.3)	1.1 (0.95-1.2)	1468 (11.4)	17 (11.6)	45 (8.9)	152 (9.4)	1.2 (0.90-1.5)	138 (9.4)	14 (9.4)

## Results

### Description of study population, selection, and exposure

Descriptive data for the study population are given in table 1. White-collar workers were more willing than blue-collar workers to participate in further studies. This was more pronounced among non-asthmatics than asthmatics. Those with high residential traffic exposure were also more willing to participate in additional studies than those with low residential traffic exposure. This difference was more pronounced among asthmatics than non-asthmatics.

In the second survey, there was an increased proportion of white-collar workers and decreased proportion of blue-collar workers answering the second survey, compared to the first survey. In the second survey, there was also a slightly higher response rate among those exposed to >19 μg/m^3 ^NO_x _, but this was not dependent on asthma status.

Description of overlap between the different traffic exposures can be seen in table 2. Residential exposure to NOx was predictive of exposure at workplace address, but less predictive of time spent outdoor in traffic. Pearson correlation between NO_x _(continous) at residential and workplace address was 0.5 (p < 0.001). The modelled concentrations of NO_x _(μg/m^3^) at residential address were: (1^st ^-3^rd ^quartile = 4.4-13), (min-max = 0.4-37), and at workplace address: (1^st ^-3^rd ^quartile = 7.1-18), (min-max = 0.8-42).

**Table 2:** Description of joint exposures

The 2^nd ^survey		NO_x _at workplace address (μg/m3)						Time outdoor in traffic/day (self-reported)				
		total	0-8	8-11	11-14	14-19	> 19	Total	0-30 min	30-1 h	1-2 h	> 2 h
NO_x _at residential address (μg/m3)	0-8	770	412(53.5%)	78(10.1%)	132(17.1%)	60(7.8%)	88(11.4%)	770	159(20.6%)	306(39.7%)	188(24.4%)	117(15.2%)
	8-11	210	30(14.3%)	44(21.0%)	59(28.1%)	32(15.2%)	45(21.4%)	210	34(16.2%)	87(41.4%)	55(26.2%)	34(16.2%)
	11-14	210	13(6.2%)	15(7.1%)	102(48.6%)	26(12.4%)	54(25.7%)	210	41(19.5%)	88(41.9%)	65(31.0%)	16(7.6%)
	14-19	161	4(2.5%)	7(4.3%)	38(23.6%)	39(24.2%)	73(45.3%)	161	29(18.0%)	65(40.4%)	41(25.5%)	26(16.1%)
												
	> 19	137	9(6.6%)	4(2.9%)	18(13.1%)	26(19.0%)	80(58.4%)	137	20(14.6%)	53(38.7%)	37(27.0%)	27(19.7%)

Percentage within row total. Exposure to residential traffic was predictive of exposure at workplace address, but less predictive of time spent outdoor in traffic. The first row shows that of those who live at a residential address with 0-8 ug NOx/m^3^, 53.5% also have a workplace address with 0-8 ug NOx/m^3 ^and 20.6% spend 0-30 min outdoor in traffic/day.

**Figure 2:**
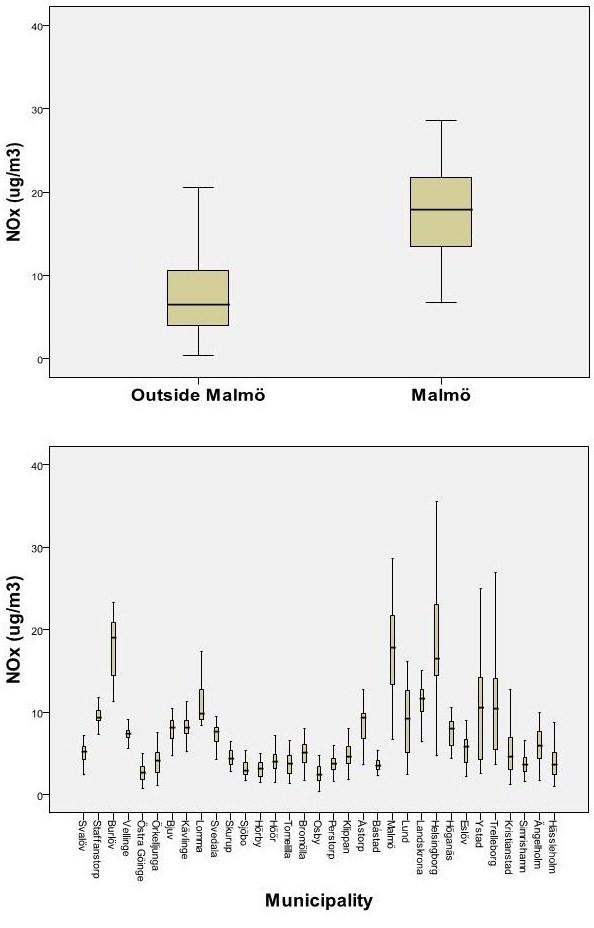
**Exposure distribution of NO_x _at residence, a) among people living in the major municipality Malmö (n = 3408 persons) vs outside (n = 19285 persons) and b) in all the 33 municipalities separately**.

The distribution of NO_x _at residential address differed between the different municipalities, with almost all in the high exposure range living in the major municipality Malmö (figure 2).

The distribution of working hours for the subjects included in the analysis of total traffic exposure was (40 hours week was considered 100% of full time): 43 persons reported working more than 100%, 984 persons worked 100%, 270 persons worked 75 to 100%, 144 persons worked 50 to 75%, and 47 persons worked less than 50%. Of those reporting asthma symptoms, 85% also reported that they used asthma medication regularly or when needed (table 3).

**Table 3:** Use of asthma medication.

			Asthma medication			
			No	Yes, when needed	Yes, regularly	Total
Asthmatic symptoms	NO	Count	2253	81	45	2379
		%	94.7%	3.4%	1.9%	100.0%
	YES	Count	68	185	200	453
		%	15.0%	40.8%	44.2%	100.0%

Of those who reported asthmatic symptoms last 12 months did 85% also report using asthma medication regularly or when needed.

### Residential traffic

Living within 50 m of a road with a traffic intensity of >10 cars/min according to GIS showed increased asthma prevalence compared to having no road within this distance (table 4). High traffic intensity within 50 and 100 m was associated with asthma symptoms last 12 months (table 4)

**Table 4:** Asthma and traffic at residential address

		The 1^st ^survey (2004)			The 2^nd ^survey (2005)				
	
Residential Address		n	Asthma n (%)	Asthma (OR)^1^	n	Asthma n (%)	Asthma (OR)^1^	Asthma symptoms n (%)	Asthma symtoms (OR)^1^
Self-report Heaviest road <50 m	0-1 cars/min	-	-	-	445	105 (23.6)	1.0	71(16.0)	1.0
	< 2 cars/min	-	-	-	1512	339 (22.4)	1.10 (0.81-1.5)	216(14.4)	0.95 (0.66-1.4)
	2-5 cars/min	-	-	-	410	113 (27.6)	1.17 (0.80-1.7)	81(19.9)	1.2 (0.76-1.8)
	6-10 cars/min	-	-	-	203	56 (27.6)	1.20 (0.74-2.0)	34(17.0)	0.79 (0.42-1.5)
	> 10 cars/min	-	-	-	258	76 (29.5)	1.5 (0.94-2.3)	48(18.5)	1.2 (0.72-2.1)

GIS Heaviest road <50 m	no heavy road	15584	1542 (9.9)	1.0	2100	494 (23.5)	1.0	316 (15.1)	1.0
	< 2 cars/min	3691	375 (10.2)	1.0 (0.90-1.2)	472	121 (25.6)	1.2 (0.89-1.6)	79 (16.8)	1.1 (0.81-1.6)
	2-5 cars/min	1555	159 (10.2)	0.95 (0.76-1.2)	216	61 (28.2)	1.2 (0.79-1.7)	39 (18.1)	1.1 (0.71-1.8)
	6-10 cars/min	307	35 (11.4)	1.0 (0.65-1.6)	34	10 (29.4)	1.0 (0.33-3.2)	7(20.6)	1.4 (0.39-5.1)
	> 10 cars/min	223	29 (13.0)	1.8 (1.1-2.8)	36	12 (33.3)	2.3 (0.99-5.2)	12 (33.3)	4.6 (2.0-10.6)

GIS Heaviest road <100 m	no heavy road	10875	1062 (9.8)	1.0	1461	330 (22.6)	1.0	215 (14.7)	1.0
	< 2 cars/min	5741	589 (10.3)	1.1 (0.92-1.2)	744	197 (26.5)	1.4 (1.1-1.7)	128 (17.4)	1.2 (0.92-1.7)
	2-5 cars/min	3309	327 (9.9)	0.96 (0.81-1.1)	462	121 (26.2)	1.2 (0.88-1.6)	75 (16.3)	1.1 (0.80-1.7)
	6-10 cars/min	894	101 (11.3)	1.2 (0.92-1.6)	119	29 (24.4)	1.4 (0.82-2.3)	14 (11.8)	0.81 (0.38-1.7)
	> 10 cars/min	541	61 (11.3)	1.3 (0.95-1.8)	72	21 (29.2)	1.6 (0.82-3.2)	21 (29.2)	2.7 (1.3-5.5)

GIS Heaviest road <250 m	no heavy road	4412	429 (9.7)	1.0	590	136 (23.1)	1.0	84 (14.2)	1.0
	< 2 cars/min	7079	698 (9.9)	1.0 (0.86-1.2)	904	225 (24.9)	1.1 (0.85-1.5)	147 (16.4)	1.1 (0.7-1.5)
	2-5 cars/min	6297	636 (10.1)	0.96 (0.82-1.1)	870	220 (25.3)	1.1 (0.83-1.5)	139 (16.1)	1.0 (0.7-1.4)
	6-10 cars/min	2100	222 (10.6)	1.1 (0.86-1.3)	298	68 (22.8)	1.2 (0.77-1.7)	42 (14.1)	1.0 (0.6-1.9)
	> 10 cars/min	1472	155 (10.5)	0.98 (0.76-1.3)	196	49 (25.0)	0.8 (0.51-1.4)	41 (20.7)	1.1 (0.6-1.9)

GIS NOx (μg/m^3 ^) (250 × 250 m)	0-8	11273	1111 (9.9)	1.0	1508	376 (24.9)	1.0	240 (16.0)	1.0
	8-11	3133	300 (9.6)	0.94 (0.79-1.1)	371	78 (21.0)	0.79 (0.56-1.1)	45 (12.3)	1.0 (0.74-1.49)
	11-14	2496	256 (10.3)	1.1 (0.93-1.3)	388	90 (23.2)	1.2 (0.86-1.6)	57 (14.8)	0.97 (0.68-1.39)
	14-19	2319	229 (9.9)	0.84 (0.69-1.0)	298	77 (25.8)	1.0 (0.73-1.5)	55 (18.4)	0.99 (0.60-1.6)
	> 19	2139	244 (11.4)	1.1 (0.93-1.4)	293	77 (26.3)	1.1 (077)	56 (19.1)	1.1 (0.60-1.9)

^1^Adjusted for age, sex, BMI, socio-economy, smoking, and occupational exposure. [OR(95%CI)]

No associations were seen with traffic intensity within 250 m or with annual mean of NO_x_.

### Traffic exposure at workplace address

No effects on asthma prevalence were seen in association with traffic at workplace address (table 5) although asthma symptoms last 12 months showed a tendency to higher prevalence with high exposure to traffic.

**Table 5:** Asthma and traffic at workplace address and during daily activities

**The 2**^**nd **^**survey**						
**WORKPLACE ADDRESS**		**n**	**Asthma n (%)**	**Asthma (OR)^1^**	**Asthma Symptoms. n (%)**	**Asthma Symptoms (OR)^1^**

Self-reported Heaviest road <50 m	0-1 cars/min	601	144 (24.0)	1.0	80 (13.4)	1.0
	2-5 cars/min	571	132 (23.1)	1.1 (0.80-1.5)	79 (14.0)	0.95 (0.66-1.4)
	6-10 cars/min	351	75 (21.4)	1.2 (0.79-1.7)	49 (14.0)	1.2 (0.76-1.8)
	> 10 cars/min	606	147 (24.3)	1.2 (0.73-1.9)	96 (15.9)	0.79 (0.42-1.5)
	Workplace varies	214	50 (23.4)	1.5 (0.93-2.7)	34 (16.0)	1.2 (0.72-2.1)

GIS Heaviest road <50 m	no heavy road	161	36 (22.4)	1.0	21 (13.2)	1.0
	< 2 cars/min	267	61 (22.8)	1.0 (0.62-1.7)	34 (12.7)	1.1 (0.55-2.1)
	2-5 cars/min	673	149 (22.1)	0.91 (0.57-1.4)	94 (14.0)	1.2 (0.65-2.1)
	6-10 cars/min	407	83 (20.4)	0.92 (0.56-1.5)	45 (11.1)	1.1 (0.58-2.0)
	> 10 cars/min	326	78 (23.9)	1.0 (0.62-1.7)	51 (15.7)	1.4 (0.72-2.6)

GIS Heaviest road <100 m	no heavy road	527	126 (23.9)	1.0	74 (14.1)	1.0
	< 2 cars/min	327	76 (23.2)	0.88 (0.61-1.3)	41 (12.5)	0.79 (0.49-1.3)
	2-5 cars/min	509	102 (20.0)	0.85 (0.61-1.2)	67 (13.2)	0.97 (0.65-1.5)
	6-10 cars/min	277	58 (20.9)	0.98 (0.66-1.5)	35 (12.7)	1.2 (0.74-1.9)
	> 10 cars/min	194	45 (23.2)	0.99 (0.63-1.5)	28 (14.5)	1.2 (0.72-2.1)

GIS Heaviest road <250 m	no heavy road	161	36 (22.4)	1.0	21 (13.2)	1.0
	< 2 cars/min	267	61 (22.8)	1.0 (0.62-1.8)	34 (12.7)	1.1 (0.55-2.1)
	2-5 cars/min	673	149 (22.1)	0.91 (0.57-1.4)	94 (14.0)	1.2 (0.65-2.1)
	6-10 cars/min	407	83 (20.4)	0.92 (0.56-1.5)	45 (11.1)	1.1 (0.58-2.0)
	> 10 cars/min	326	78 (23.9)	1.0 (0.62-1.7)	51 (15.7)	1.4 (0.72-2.6)

GIS NO_x _(μg/m^3) ^(250 × 250 m)	0-8	558	129 (23.1)	1.0	70 (12.6)	1.0
	8-11	163	34 (20.9)	0.88 (0.55-1.4)	23 (14.1)	1.1 (0.65-2.0)
	11-14	455	94 (20.7)	0.91 (0.65-1.3)	56 (12.4)	0.99 (0.64-1.5)
	14-19	227	48 (21.1)	1.0 (0.68-1.5)	27 (11.9)	1.2 (0.71-2.0)
	> 19	431	102 (23.7)	0.98 (0.70-1.4)	69 (16.1)	1.3 (0.88-2.0)

**DAILY ACTIVITIES**		**n**	**Asthma n (%)**	**Asthma (OR)^1^**	**Asthma symptoms n (%)**	**Asthma Symptoms n (%)**

Time outdoor in traffic/day	0-30 min	622	134 (21.5)	1.0	79 (12.8)	1.0
	30 min-1 h	1066	248 (23.3)	1.1 (0.8-1.4)	159 (15.1)	1.2 (0.83-1.7)
	1-2 h	715	194 (27.1)	1.1 (0.8-1.5)	121 (17.0)	1.4 (0.91-2.0)
	> 2 h	453	121 (26.7)	1.0 (0.7-1.4)	92 (20.4)	1.3 (0.83-2.0)

Commuting time to work	< 15 min	881	211 (24.0)	1.0	117 (13.4)	1.0
	15-30 min	915	207 (22.6)	0.90 (0.70-1.1)	140 (15.4)	1.1 (0.84-1.5)
	30 min-1 h	408	99 (24.3)	1.0 (0.73-1.4)	60 (14.8)	1.2 (0.78-1.7)
	> 1 h	129	29 (22.5)	0.77 (0.45-1.33)	18 (14.2)	0.92 (0.47-1.8)

^1^Adjusted for age, sex, BMI, socio-economy, smoking, and occupational exposure. [OR(95%CI)]

### Traffic exposure during daily activities

No effects on asthma were seen from self-reported daily time spent in traffic or commuting time to and through work, after adjustment for confounders (adjusted estimates in table 5), although time spent in traffic showed an unadjusted association with asthma symptoms, 1-2 h in traffic (OR = 1.4 (1.0-1.9)) and >2 h in traffic (OR = 1.8(1.3-2.4)) compared to 0-30 min in traffic.

### Accounting for total traffic exposure

Combining traffic exposure at residential address, with workplace address and self-reported daily time spent in traffic did not increase the association with asthma (table 6).

**Table 6:** Total traffic exposure.

**The 2**^**nd **^**survey**						
**COMBINED EXPOSURE**	**n**	**Asthma, n (%)**	**Asthma, n (%)**	**Asthma OR^1^**	**Asthma symptoms, n (%)**	**Asthma symptoms (OR)^1^**

Total exposure^2 ^*C = 30*	1^st^	298	72(24.2%)	1.00	41(13.8%)	1.00
	2^nd^	298	68(22.8%)	0.90 (0.61-1.35)	32(10.8%)	0.70(0.41-1.18)
	3^rd^	297	59(19.9%)	0.76 (0.51-1.15)	43(14.5%)	1.09(0.67-1.77)
	4^th^	298	65(21.8%)	0.87 (0.58-1.31)	41(13.8%)	1.09(0.66-1.79)
	5^th^	297	70(23.6%)	0.96 (0.64-1.44)	48(16.2%)	1.28(0.79-2.08)

Total exposure^2 ^*C = 300*	1^st^	298	67(22.5%)	1.00	35(11.8%)	1.00
	2^nd^	298	67/22.5%)	1.02 (0.68-1.53)	35(11.8%)	1.06(0.63-1.79)
	3^rd^	297	69(23.2%)	1.00 (0.66-1.50)	48(16.2%)	1.50(0.91-2.48)
	4^th^	298	65(21.8%)	0.88 (0.58-1.34)	45(15.2%)	1.33(0.79-2.21)
	5^th^	297	66(22.2%)	0.88 (0.58-1.33)	42(14.1%)	1.18(0.71-1.99)

Residential + workplace Address^2^	1^st^	298	73(24.5%)	1.00	41(13.8%)	1.00
	2^nd^	298	69(23.2%)	0.91 (0.61-1.35)	36(12.1%)	0.89(0.53-1.47)
	3^rd^	297	54(18.2%)	0.64 (0.42-0.97)	36(12.2%)	0.87(0.52-1.44)
	4^th^	298	69(23.2%)	0.98 (0.66-1.46)	46(15.5%)	1.31(0.81-2.12)
	5^th^	297	69(23.2%)	0.94 (0.63-1.41)	46(15.5%)	1.27(0.78-2.07)

Workplace Address	1^st^	298	74(24.8%)	1.00	40(13.5%)	1.00
	2^nd^	298	64(21.5%)	0.81 (0.54-1.22)	41(13.8%)	1.12(0.68-1.85)
	3^rd^	297	66(22.2%)	0.87 (0.58-1.31)	41(13.8%)	1.11(0.67-1.85)
	4^th^	298	67/22.5%)	0.92 (0.62-1.37)	40(13.5%)	1.14(0.70-1.88)
	5^th^	297	63(21.2%)	0.77 (0.51-1.16)	43(14.6%)	1.19(0.72-1.96)

Residential Address	1^st^	298	71(23.8%)	1.00	41(13.8%)	1.00
	2^nd^	298	70(23.5%)	0.90 (0.60-1.34)	35(11.8%)	0.80(0.48-1.33)
	3^rd^	297	58(19.5%)	0.78 (0.52-1.18)	41(13.9%)	1.08(0.66-1.75)
	4^th^	298	66(22.1%)	0.91 (0.60-1.36)	40(13.4%)	1.09(0.66-1.80)
	5^th^	297	69(23.2%)	0.96 (0.64-1.44)	48(16.2%)	1.31(0.81-2.13)

^1^Adjusted for age, sex, BMI, socio-economy, smoking, and occupational exposure. [OR(95%CI)]. ^2 ^Total exposure assessment (residential address + workplace address + time in traffic) is explained in methods section. The estimate based on only residential + workplace address is also time-weighted. C is the exposure dose time in traffic is hypothesized to contribute.

The association between traffic and asthma is not stronger when combining total exposure compared to using only residential exposure. Using quantiles, i.e holding the number of individuals fixed in each category, the changes in estimates reflects individuals moving between the low/high categories depending on what exposures (residential address, workplace address, time outdoor in traffic) that are combined to estimate high vs low traffic exposure.

Adjusting the association between asthma and traffic intensity at residential address (within 100 m), for traffic intensity at work-address(within 100 m), and daily time spent in traffic, with and without adjustment for other confounders, did not change the estimate at residence noticeably (< 10%).

Similarly, associations with traffic intensity at workplace address (within 100 m) and time spent in traffic, were robust to adjustment for other traffic exposures.

### Restricted analyses

The effects on asthma prevalence from traffic were stronger and statistically significant when limiting to people living on their current address >5 years (data not shown). Restricting the analysis to asthma cases which also had doctors diagnosis of asthma did not significantly alter the estimates. Restricting the analyses to subgroups of asthmatics who had answered "Yes, minor symptoms" or "Yes, severe symptoms" (compared to "No asthma") did not significantly alter the estimates. Use of asthma medication was associated with having a road with a traffic intensity of >10 cars/min, within 50 m (adj. OR = 3.24(1.39-7.58) and within 100 m (adj. OR = 2.07(1.01-4.27) of residence, compared to having no road within the same distance, but use of asthma medication was not associated with the other traffic exposures.

## Discussion

Living in close proximity to traffic was associated with increased prevalence of asthma and asthma symptoms last 12 months. No statistically significant effects were seen from traffic exposure at workplace address, daily time spent in traffic, or commuting time to work, after adjustment for potential confounders. A combined exposure estimate did not give higher association with asthma.

### Discussion of exposure assessment

This is to our knowledge the first epidemiological study on asthma to use GIS not only to estimate traffic at residential address but also at workplace address and with information about commuting time to work or other outdoor time in traffic. However, while this more complete exposure information could be expected to strengthen any association with asthma, this was not found in this study.

A potential reason that no significant adverse effect was seen on workplace address could be if misclassification of exposure, due to invalid geocoding, was larger for workplace address. Since geocoding for the workplace address was made for the exact address, the geocoding technique in itself is not likely to be the reason for no association. However, if the study subjects are not stationary at their work location, or the company address might refer to larger commercial areas or buildings there might be little association between the personal exposure and the outdoor-indoor levels for that location. Exposure estimates at the residential addresses might on the other hand have inaccuracies due to imprecise geocoding since individuals are positioned at the centre of their real estates. In urban areas there might therefore be substantial misplacement for individuals living in large family-housing, or for large estates with vast land areas in the rural areas. It is well known that geocoding error generally gives conservative estimates [21], as does exposure misclassification in general if not related to disease.

It should be noted that effects of traffic on asthma symptoms were indicated at workplace addresses, but the effect estimates were lower than at residential address, and not statistically significant.

Since the associations between traffic-related air pollution and asthma generally shows distance-dependent relationship with strongest effects on asthma from living within 50 m of roads, and with sharp decline of many air pollutants within 30-150 m, a modelled resolution on NO_x _of 250 × 250 m might be too low to detect any effects from traffic. This must be weighted against the fact that a higher spatial resolution may not be meaningful considering the likely location uncertainty of workplace address.

An effect from daily time spent in traffic on asthma symptoms was indicated in unadjusted estimates, but not after adjustment for confounders. Exposure studies and simulations studies have shown that personal NO_x _dose *per se *is only marginally influenced by commuting time [11], but if NO_x _is seen as a proxy for NO and ultrafine particles, or other pipe-exhausts, the contribution from time in traffic outdoor at street-level i.e in congested traffic, may be many times higher and very influential of total exposure. In this study we regarded NO_x _as a proxy for traffic pollution and treated use of gas stove as a potential confounder rather than exposure. When calculating the contribution of "time in traffic" to total exposure, we let the "dosecontribution" vary between 30 μg/m^3 ^and a more extreme scenario of 300 μg/m^3^, but this did not give a stronger association with asthma, although some of the asthma cases were moved from the lowest to a higher exposure category.

The major source of exposure misclassification may be the cross-sectional study character, especially for asthma prevalence, which showed an increased association with traffic when analysis was restricted to subjects which had been living at least 5 years at current address. Although asthma may start in adult age, most asthma begin in childhood [22], hence, a cross-sectional study in adults may poorly reflect retrospective exposure. This however should less affect the results for asthma symptoms last 12 months, a condition which is better related to current exposure, but may have different etiology and be affected differently by air pollution [23].

Since air pollution is well known to trigger symptoms [1,23], (even if it is less certain if it contributes to the development of asthma), asthmatics may be more likely to move away from than towards traffic. Therefore a migrational bias is most likely to decrease the effects on asthma prevalence and asthma symptoms. It is also likely that the large proportion (44%) who regularly used asthma medication further would diminish the association between traffic and asthma symptoms, especially since people living closing to roads had a higher prevalence of asthma medication. In conclusion, cross-sectional studies need to be confirmed by prospective studies, not only to establish the casual link, but also to measure the true burden of disease from traffic.

Since this study was conducted in an area with low levels of air pollution in a European perspective, high exposure to traffic was rare and the study was slightly underpowered to estimate effects from residential traffic at traffic levels which has previously shown to be related to effects. This also hindered any further analysis of effect modifications by other risk factors than traffic. Pooling of exposure groups would not help since only the highest exposure groups showed a relation to traffic, thus pooling would severely dilute the effects.

### Discussion of potential confounding and selection bias

A strength of the study was the large number of potential confounder information which was collected, such as BMI [24], occupational exposure [25], and presence of indoor dampness and mould [26], which are known risk factors for adult asthma and often associated with socio-economic status of the neighbourhood. Socio-economic status (SEI), with the classification system used in this study, has in Sweden shown an association with asthma incidence in recent years [27]. Confounder adjustment slightly increased the effect estimates for residential address, suggesting that competing risk factors sometimes dilute the effects from traffic, something we have previously suggested [17]. A weakness was that we did not have more detailed data on triggers for asthmatic symptoms, since we previously have observed a association between traffic and asthma triggered by pollen and furred animals, but not with asthma triggered by other factors [28]. Degree of confounding (measured or unmeasured) is not likely to be directly generalizable between studies since the association between covariates such as socio-economic status and air pollution (NO_x_) has been shown to be reversed depending on area in Scania [29]. Confounding is better controlled for with respect to asthma symptoms than to asthma prevalence in this study, since we had information about current but not past exposure to risk factors.

The effect estimates for residential traffic were stronger in the case-control study than in the first survey, indicating potential selection bias. In previous public health surveys in the region it has been shown that the response rate is dependent on geographical strata [30]. It is thus not unlikely that selection bias can have occurred, however the objective exposure assessments used in this study is a true advantage. Ideally, since this study was sampled on geographical strata, an analysis conditional on geographical stratum might have increased the validity. This was however not possible since exposure ranges were not comparable between the different stratas/communities (figure 2). This also excluded the possibility to use a dummy variable for urban/rural areas to adjust for potential residual urban-rural confounding. It should be noted that accounting for total traffic exposure could further have strengthened any residual urban-rural confounding by comparing people who are both working and living in rural environments, with people who are both working and living in urban environments.

### Results discussion

To our knowledge, all previous studies on adult asthma prevalence have only estimated traffic exposure at residential address. A previous cross-sectional study in southern Sweden found asthma triggered by allergic factors to be associated with high traffic intensity within 100 m of residence, and with modelled *NO_x _*> 19 μg/m^3 ^[17,28]. A cross-sectional study in northern Sweden found that asthmatic symptoms increased significantly with modelled NO_2_-concentrations and self-reported heavy vehicles outside the kitchen window [31]. A Swedish case-control study found measured home outdoor NO_2 _(min-max: 0-29 μg/m^3^) to be associated with asthma incidence among atopics [5]. The Swedish cities in the Nordic Rhine study found modelled NO_2 _to be associated with incident asthma (OR = 1.5, 95% CI 1.0-2.4, per 10 μg/m^3^) (min-max: 3.3-46 μg/m^3^) [6].

A few European cohort studies have supported that traffic pollution increases asthma incidence in adults: The ECRHS study found an association between modelled NO_2 _and increased asthma incidence (OR 1.4; 95% CI 1.0-2.0, per 10 μg/m^3^) [3], The SAPALDIA study found that asthma incidence was associated with modelled change in TPM_10_, hazard ratio (1.3, 95%CI: 1.1 - 1.6 per 1 μg/m^3 ^change) [4]

The results from other Swedish studies support that asthma symptoms are affected at relatively low levels of air pollution. Cohort studies in adults, although still few, also supports that the association between traffic exposure and asthma prevalence observed in this cross-sectional study may reflect a true increase in asthma incidence when living close to traffic.

However, if the most recent studies support the association between air pollution and asthma, the relation with asthma incidence is not fully settled and there are also a few recent negative studies in adults [32,33], and some cohorts in children [34].

There are two studies in children which have investigated the effects of traffic at both home and school, on asthma. McConnell et al found an increased hazard ratio when combining traffic-related pollutants at school-and residential address, on new-onset asthma, compared to the independent effects [12]. The other study by Kim et al make a reservation that the study was not designed for independent assessment of exposure at school- and residential address, and the sample size was insufficient to properly do so, but they report that they found a slight attenuation of effects on current asthma from residential traffic pollution when adding both residential and school exposure in the same model [13].

In our study, effects at workplace address in the highest exposure categories were statistically insignificant partly because lack of power to confirm small effect estimates. Further studies in areas where high levels of air pollution is rare, should consider to strongly oversample exposed subjects in relevant exposure ranges and population groups.

However, the lack of power can not explain that the association did not get stronger for total exposure. Alhough our lack of statistically significant association with traffic at workplace address and time spent in traffic may be due to misclassification of exposure, it may also indicate that residence is still the most influential exposure determinant of traffic exposure among adults.

## Conclusions

Living within 50 m of a road with high traffic intensity was associated with higher prevalence of asthma and asthma symptoms last 12 months. No statistically significant effects were seen from traffic exposure at work-address, daily time spent in traffic, or commuting time to and through work. A combined total exposure estimate did not give a stronger association with asthma prevalence or asthma symptoms.

## Competing interests

The authors declare that they have no competing interests.

## Authors' contributions

AL wrote the main part part of the manuscript and conducted the statistical analyses. ES provided GIS-data and made revisions on draft. JB and KJ designed and conducted the surveys and made revisions on drafts. All authors read and approved the final manuscript.

## Pre-publication history

The pre-publication history for this paper can be accessed here:

http://www.biomedcentral.com/1471-2458/10/716/prepub

## Supplementary Material

Additional file 1 **Survey1_2004_Swedish original**. The Swedish original questionnaire for the first survey (2004). Click here for file

Additional file 2 **Survey1_2004_English translation**. English translation of the first survey questionnaire (2004). Click here for file

Additional file 3 **Survey2_2005_Swedish orginal**. The Swedish original questionnaire for the second survey (2005). Click here for file

Additional file 4 **Survey2_2005_English translation**. English translation of the second survey questionnaire (2005). Click here for file

## References

[B1] WHO Air Quality Guidelines Global Update 2005

[B2] Salam MT I T Gilliland FD Recent evidence for adverse effects of residential proximity to traffic sources on asthma Current opinion in pulmonary medicine 2008 14 1 3 8 10.1097/MCP.0b013e3282f1987a 18043269

[B3] Jacquemin B S J Forsberg B Aguilera I Briggs D Garcia-Esteban R Götschi T Heinrich J Järvholm B Jarvis D Viennau D Künzli N Home outdoor NO2 and new onset of self-reported asthma in adults Epidemiology 2009 20 1 119 26 10.1097/EDE.0b013e3181886e76 18923331

[B4] Kûnzli N B P Liu LJ Garcia-Esteban R Schindler C Gerbase MW Sunyer J Keidal D Rochat T Swiss Cohort Study on Air Pollution and Lund Disease in Adults Traffic-related air pollution correlates with adult-onset asthma among never-smokers Thorax 2009 64 8 664 70 10.1136/thx.2008.110031 19359271

[B5] Modig L J B Rönnmark E Nyström L Lundbäck B Andersson C Forsberg B Vehicle exposure in an incident case-control study of adult asthma European Respiratory Journal 2006 28 1 75 81 10.1183/09031936.06.00071505 16540504

[B6] Modig L T K Janson C Jarvholm B Forsberg B Vehicle exhaust outside the home and onset of asthma among adults European Respiratory Journal 2009 33 6 1261 67 10.1183/09031936.00101108 19251785

[B7] Jerrett M Does traffic-related air pollution contribute to respiratory diesase formation in children? European Respiratory Journal 2007 29 825 826 10.1183/09031936.00022607 17470616

[B8] Mckone TE R P Özkaynak H Exposure information in environmental health research: Current opportunities and future directions for particulate matter, ozone, and toxic air pollutants Journal of Exposure Science and Environmental Epidemiology 2009 19 30 34 10.1038/jes.2008.3 18385670

[B9] Greenland S The effect of misclassfication in the presence of covariates American Journal of Epidemiology 1980 112 4 564 9 7424903 10.1093/oxfordjournals.aje.a113025

[B10] Briggs D The role of GIS: Coping with space (and time) in air pollution exposure assessment Journal of Toxicology and Environmental Health, Part A 2005 68 1243 61 10.1080/15287390590936094 16024500

[B11] Setton EM K C Cloutier-Fisher D Hystad PW Spatial variations in estimated chronic exposure to traffic-related air pollution in working populations: A simulation International Journal of Health Geographics 2008 7 39 10.1186/1476-072X-7-39 18638398 PMC2515287

[B12] McConnell R I T Shankardass K Jerrett M Lurmann F Gilliland F Gauderman J Avol E Kûnzli N Yao L Peters J Berhane K Childhood incident asthma and traffic-related air pollution at home and school Environmental Health Perspectives 2010 118 7 1021 26 10.1289/ehp.0901232 20371422 PMC2920902

[B13] Kim JJ H K Adams S Smorodinsky S Hoats A Malig B Lipsett M Ostro B Residential traffic and children's respiratory health Environmental health perspectives 2008 116 9 1274 9 10.1289/ehp.10735 18795175 PMC2535634

[B14] Ali SM C B Merlo J Rosvall M Wamala S Lindström M Gender differences in daily smoking prevalence in different age strata: a population-based study in southern Sweden Scandinavian Journal of Public Health 2009 37 2 146 52 10.1177/1403494808100274 19141546

[B15] Gustafsson S ed Uppbyggnad och validering av emissionsdatabas avseende luftföroreningar för Skåne med basår 2001 [A geographical and temporal high resolution emission database for dispersion modelling of environmental NOX in Southern Sweden] The Department of Physical Geography and Ecosystem Analysis 2007 Lund University: Lund

[B16] Stroh E The use of GIS in Exposure-Response Studies The Department of Physical Geography and Ecosystem Analysis 2006 Lund University: Lund

[B17] Lindgren A S E Montnémery P Nihlén U Jakobsson K Axmon A Traffic-related air pollution associated with prevalence of asthma and COPD/chronic bronchitis. A cross-sectional study in Southern Sweden International Journal of Health Geographics 2009 8 2 10.1186/1476-072X-8-2 19154599 PMC2649061

[B18] Löhndahl J M A Swietlicki E Bräuner EV Ketzel M Pagels J Loft S Experimentally determined human respiratory tract deposition of airborne particles at a busy street Environmental Science & Technology 2009 1 43 13 10.1021/es803029b 19673248

[B19] Statistics-Sweden The Socio-economic Classification of Occupation Stockholm 1982

[B20] Matheson MC B G Raven J Sim MR Kromhaut H Vermeulen R Johns DP Walters EH Abramson MJ Biological dust exposure in the workplace is a risk factor for chronic obstrucitve pulmonary disease Thorax 2005 60 8 645 51 10.1136/thx.2004.035170 16061705 PMC1747486

[B21] PA Z Influence of geocoding quality on environmental exposure assessment of children living near high traffic roads BMC Public Health 2007 7 37 10.1186/1471-2458-7-37 17367533 PMC1838415

[B22] Bel E Clinical phenotypes of asthma Current opinion in pulmonary medicine 2004 10 1 44 50 10.1097/00063198-200401000-00008 14749605

[B23] Peden D The epidemiology and genetics of asthma risk associated with air pollution The Journal of Allergy and Clinical Immunology 2005 115 2 213 19 10.1016/j.jaci.2004.12.003 15696070

[B24] Chen Y D R Jiang Y The association between obesity and asthma is stronger in nonallergic than allergic adults Chest 2006 130 890 895 10.1378/chest.130.3.890 16963691

[B25] Torén K B P Asthma caused by occupational exposures is common - A systematic analysis of estimates of the population-attributable fraction BMC Pulmonary Medicine 2009 9 7 10.1186/1471-2466-9-7 19178702 PMC2642762

[B26] Jaakkola MS N H Piipari R Uitti J Laitinen J Karjalainen A Hahtola P Jaakkola JJK Indoor Dampness and Molds and Development of Adult-Onset Asthma: A Population-Based Incident Case-Control Study Environmental Health Perspective 2002 110 543 547 10.1289/ehp.02110543 PMC1240846 12003761

[B27] Bråbäck L H A Rasmussen F Social class in asthma and allergic rhinitis: a national cohort study over three decades European Respiratory Journal 2005 26 1064 1068 10.1183/09031936.05.00022105 16319336

[B28] Lindgren A S E Nihlén U Montnémery P Axmon A Jakobsson K Traffic exposure associated with allergic asthma and allergic rhinitis in adults. A cross-sectional study in southern Sweden International Journal of Health Geographics 2009 8 25 10.1186/1476-072X-8-25 19419561 PMC2687434

[B29] Stroh E O A Gustafsson S Pilesjö P Harrie L Strömberg U Jakobsson K Are associations between socio-economic characteristics and exposure to air pollution a question of study area size? An example from Scania, Sweden 2005 4 30 10.1186/1476-072X-4-30 PMC1315343 16288656

[B30] Carlsson F M J Lindström M Ostergren PO Lithman T Representativity of a postal health questionnaire survey in Sweden, with special reference to ethnic differences in participation Scandinavian Journal of Public Health 2006 34 2 132 9 10.1080/14034940510032284 16581705

[B31] Modig L F B Perceived annoyance and asthmatic symptoms in relation to vehicle exhaust levels outside home: a cross-sectional study Environmental health 2007 6 29 10.1186/1476-069X-6-29 17903240 PMC2048499

[B32] Pujades-Rodriguez M L S Mckeever T Britton J Venn A Effect of living close to a main road on asthma, allergy, lung function and chronic obstructive pulmonary disease Occupational and Environmental Medicine 2009 66 679 684 10.1136/oem.2008.043885 19770354

[B33] Mar Pujades-Rodriguez T M Sarah Lewis Duncan Whyatt John Britton Andrea Venn Effect of traffic pollution on respiratory and allergic disease in adults: cross-sectional and longitudinal analysis BMC Pulmonary Medicine 2009 9 42 10.1186/1471-2466-9-42 PMC2744653 19703291

[B34] Oftedahl B Nystad W Brunekreef B Nafstad P Long-term traffic-related exposures and asthma onset in schoolchildren in oslo, norway Environmental health perspectives 2009 117 5 839 44 19478970 10.1289/ehp.11491 PMC2685850

