# Supplementary material for: Adult asthma and traffic exposure at residential address, workplace address, and self-reported daily time outdoor in traffic: A two-stage case-control study
Source: BMC Public Health. 2010 Nov 22;10:716. doi: 10.1186/1471-2458-10-716 (PMC3003254; doi:10.1186/1471-2458-10-716)
Supplement: Additional file 1 — Survey1_2004_Swedish original. The Swedish original questionnaire for the first survey (2004). [file 1471-2458-10-716-S1.PDF]

Folkhälsa i Skåne 2004

Var med och påverka utformningen av den framtida sjukvården i Skåne!

Hur kan vi förbättra det förebyggande arbetet?

Hur kan hälsan förbättras i Skåne?

Du är en av de personer i Skåne som blivit inbjuden till att delta i denna undersökning. Syftet är att öka kunskapen om skåningarnas hälsa och miljö. Det frågeformulär du fått innehåller bland annat frågor om ditt hälsotillstånd och viktiga faktorer av betydelse för hälsan.

Varför är det viktigt att just du svarar på enkäten?
Den här undersökningen gör vi för att få en aktuell bild av skåningarnas hälsa och vårdkontakter och av levnads- och miljöförhållanden som har betydelse för den framtida folkhälsan i Skåne. Ditt deltagande är frivilligt. För att resultaten ska bli rättvisande är det viktigt att så många som möjligt svarar på enkäten. När du besvarar frågeformuläret gör du alla andras medverkan mer meningsfull. Dina svar kommer att hjälpa oss i vårt arbete med att utforma olika hälsofrämjande och sjukdomsförebyggande åtgärder och att förbättra hälso- och sjukvården i Skåne. Därför är ditt svar mycket viktigt!

Vad händer med dina svar?
Givetvis kommer din identitet och alla uppgifter om dig att skyddas noga och vara sekretessbelagda. All information som vi sammanställer kommer att redovisas i tabeller där det är helt omöjligt att identifiera någon enskild individ.

Undrar du över något?
Socialmedicinska enheten är ansvarig för undersökningen, men har gett Statistiska centralbyrån uppdraget att utföra utskick, insamling av frågeformulären och sammanställningar. Om det är något du vill tala med oss om i samband med ifyllandet av formuläret eller om undersökningen i övrigt är du mycket välkommen att ringa något av de nummer som finns angivna på nästa sida.

Med vänliga hälsningar

Kent Karlsson
Fil.dr., Strateg
Hälso- och sjukvårdsledningen
Region Skåne

Farhad Ali Khan
Hälsoplanerare, Projektledare
Socialmedicinska enheten
Universitetssjukhuset MAS

Per-Olof Östergren
Professor, Verksamhetschef
Socialmedicinska enheten
Universitetssjukhuset MAS

Ett varmt tack på förhand för Din medverkan!

MER FAKTA OM UNDERSÖKNINGEN

För att minska antalet frågor i enkäten kommer folkbokföringsuppgifter såsom ålder, födelseland och medborgarskap att hämtas från SCB:s register över totalbefolkningen (RTB). Från andra register på SCB kommer uppgifter om utbildningsnivå, sysselsättning och inkomst att hämtas. Dessutom lägger SCB på en kod som möjliggör olika geografiska indelningar. Uppgifter om läkarbesök, sjukhusvistelser och eventuell sjukskrivning hämtas från landstingets databaser och Riksförsäkringsverket.

Dina svar skyddas av sekretesslagen (SFS 1980:100, 9 kap. 4§) och personuppgiftslagen. Det betyder att alla som arbetar med undersökningen har tystnadsplikt och att de insamlade uppgifterna redovisas i tabeller där ingen enskilds svar kan utläsas. Numret högst upp på blanketten är till för att SCB under insamlingen ska se vilka som svarat och vilka som ska få en påminnelse.

Alla identitetsuppgifter avlägsnas innan SCB lämnar över materialet till Socialmedicinska enheten. För att vi ska kunna följa upp hälsoläget i framtiden kommer SCB att bevara en ”nyckel” mellan namn och enkätens löpnummer i tio år framåt. Det är enbart berörda inom SCB som kommer att ha tillgång till dessa uppgifter.

Har du frågor om själva undersökningen (bakgrund, syfte, hur du ska besvara frågorna etc.) är du välkommen att ringa till: Viveca Flodén, Socialmedicinska enheten, Universitetssjukhuset MAS, tfn 040-33 28 74 kl. 13.00-14.30 eller skicka e-post till fhs2004@skane.se.

Har du frågor om själva datainsamlingen är du välkommen att ringa till: SCB, tfn 019-17 60 65 eller skicka e-post till fhs2004@scb.se.

Så här fyller du i enkäten

Att besvara frågorna tar ca 20-30 minuter. Enkätsvaren registreras maskinellt och därför är det viktigt att de är ifyllda på rätt sätt.

- Använd en bra kulspetspenna med svart eller mörkblå färg. Undvik blyerts.

- Sätt ett kryss i de rutor du tycker stämmer bäst för dig. Markera om möjligt innanför rutorna.

Så här: ☒

INTE så här: ☒

- Om du råkar kryssa fel fyller du i hela den rutan i färg.

Så här: ■

Då kan inte maskinen läsa den. Kryssa sedan i rätt ruta.

Svarstid

Vi sätter stort värde på om vi kan få ditt ifyllda frågeformulär inom en vecka.

Svarskuvert och porto

Skicka enkäten i bifogat kuvert. Portot är redan betalt.

Tack för din medverkan!

Hälsa

Fråga 1 Hur bedömer Du Ditt nuvarande hälso-tillstånd i allmänhet?

1 ☐ Mycket bra
2 ☐ Bra
3 ☐ Någorlunda
4 ☐ Dåligt
5 ☐ Mycket dåligt

Fråga 2 Hur lång är Du? *Hela cm.*

☐☐☐ cm

Fråga 3 Hur mycket väger Du? *Hela kg.*

☐☐☐ kg

Fråga 4 Har Du under de senaste 14 dagarna varit besvärad av något av de symtom eller obehag som nämns nedan? *Sätt ett kryss på varje rad!*

	I hög grad<br>1	I någon mån<br>2	I liten mån<br>3	Över huvud taget inte<br>4
a) Värk i skuldror, nacke eller axlar	☐	☐	☐	☐
b) Ryggsmärtor, ryggvärk, höftsmärtor eller ischias	☐	☐	☐	☐
c) Värk eller smärtor i händer, armbågar, ben eller knän	☐	☐	☐	☐
d) Huvudvärk eller migrän	☐	☐	☐	☐
e) Ängslan, oro eller ångest	☐	☐	☐	☐
f) Trötthet	☐	☐	☐	☐
g) Sömnsvårigheter	☐	☐	☐	☐
h) Eksem eller hudutslag	☐	☐	☐	☐
i) Öronsus (tinnitus)	☐	☐	☐	☐
j) Inkontinens (urinläckage)	☐	☐	☐	☐
k) Återkommande mag-/tarmbesvär	☐	☐	☐	☐

Fråga 5 Har Du något/några av följande hälsoproblem?

Sätt ett kryss på varje rad!	Nej<br>1	Ja, men inga besvär<br>2	Ja, lätta besvär<br>3	Ja, svåra besvär<br>4
a) Allergi	☐	☐	☐	☐
b) Astma	☐	☐	☐	☐
c) Diabetes	☐	☐	☐	☐
d) Högt blodtryck	☐	☐	☐	☐

Fråga 6 **a) Har Du någon gång under de senaste 14 dagarna p.g.a. någon sjukdom, skada, eller annan åkomma varit förhindrad att utföra Dina vanliga sysslor (t.ex. arbete inom eller utanför hemmet, fritidsaktiviteter eller liknande)?**

1 ☐ Ja

2 ☐ Nej ———> **Gå till fråga 7**

b) Om JA: Är dessa besvär av en mera bestående karaktär? Med bestående menas att de funnits eller förväntas vara 6 månader eller mera.

1 ☐ Ja

2 ☐ Nej

Om du inte arbetar gå till fråga 9.

Fråga 7 **a) Har Du varit tvungen att stanna hemma från arbete på grund av sjukdom, skada eller andra besvär under det senaste året?**

1 ☐ Nej, inte alls det senaste året→**Gå till fråga 8**

2 ☐ Ja

b) Om JA: Hur många arbetsdagar?
Räkna bara arbetsdagar!

☐☐☐ arbetsdagar

Fråga 8 **a) Har Du varit tvungen att stanna hemma från arbete på grund av sjukdom, skada eller andra besvär under de senaste två veckorna?**

1 ☐ Nej, inte alls de senaste två veckorna

2 ☐ Ja

b) Om JA: Hur många arbetsdagar?
Räkna bara arbetsdagar!

☐☐ arbetsdagar

Fråga 9 **Har Du någon långvarig sjukdom, besvär efter olycksfall, något handikapp eller annan svaghet?**

1 ☐ Ja

2 ☐ Nej

Fråga 10 **Har Du någonsin på grund av sjukdom, skada eller besvär varit tvungen att sluta ett arbete, byta till annat arbete eller andra arbetsuppgifter?**

1 ☐ Ja, bytt arbete/arbetsuppgifter

2 ☐ Ja, slutat ett arbete

3 ☐ Ja, först bytt arbete/arbetsuppgifter och sedan slutat arbeta

4 ☐ Nej

Fråga 11 Frågorna här handlar om hur Du känner Dig och hur Du haft det under de senaste fyra veckorna. Ange för varje fråga det svarsalternativ som bäst beskriver hur Du känt Dig.

Hur stor del av tiden under de senaste fyra veckorna ...	Hela tiden<br>1	Största delen av tiden<br>2	En hel del av tiden<br>3	En del av tiden<br>4	Lite av av tiden<br>5	Inget av tiden<br>6
a) har Du känt Dig riktigt pigg och stark?	☐	☐	☐	☐	☐	☐
b) har Du känt Dig mycket nervös?	☐	☐	☐	☐	☐	☐
c) har Du känt Dig så nedstämd att ingenting har kunnat muntra upp Dig?	☐	☐	☐	☐	☐	☐
d) har Du känt Dig lugn och harmonisk?	☐	☐	☐	☐	☐	☐
e) har Du varit full av energi?	☐	☐	☐	☐	☐	☐
f) har Du känt Dig dyster och ledsen?	☐	☐	☐	☐	☐	☐
g) har Du känt Dig utsliten?	☐	☐	☐	☐	☐	☐
h) har Du känt Dig glad och lycklig?	☐	☐	☐	☐	☐	☐
i) har Du känt Dig trött?	☐	☐	☐	☐	☐	☐

Läkemedel

Fråga 12 Har Du under de tre senaste månaderna använt något av följande läkemedel eller preparat?

Sätt ett kryss på varje rad!	Nej<br>1	Ja, senaste tre månaderna<br>2	Ja, senaste 14 dagarna<br>3
a) Blodtryckssänkande medicin	☐	☐	☐
b) Medicin mot astma/allergi	☐	☐	☐
c) Smärtstillande medel, köpt utan recept	☐	☐	☐
d) Smärtstillande medel, köpt med recept	☐	☐	☐
e) Antibiotika	☐	☐	☐
f) Sömnmedel	☐	☐	☐
g) Nervlugnande medicin	☐	☐	☐
h) Medel mot depression	☐	☐	☐
i) Magsårsmedicin	☐	☐	☐
j) Östrogenpreparat	☐	☐	☐
k) Vitaminer/Mineraler	☐	☐	☐
l) Naturläkemedel	☐	☐	☐
m) Annat ____________	☐	☐	☐

Fråga 13 Användning av läkemedel

Sätt ett kryss på varje rad!	Ja<br>1	Nej<br>2	Tar ej medicin<br>3
a) Händer det att Du glömmer att ta Din medicin?	☐	☐	☐
b) Är Du alltid noga med att ta Din medicin?	☐	☐	☐
c) Om Du känner Dig bättre, händer det då att Du låter bli att ta Din medicin?	☐	☐	☐
d) Om Du känner Dig sämre när Du tar Din medicin händer det då att Du låter bli att ta den?	☐	☐	☐

Fråga 14 a) Har Du under de tre senaste månaderna någon gång fått recept utskrivet, men inte hämtat ut det på apoteket?

1 ☐ Ja, en gång

2 ☐ Ja, flera gånger

3 ☐ Nej ———> **Gå till fråga 15**

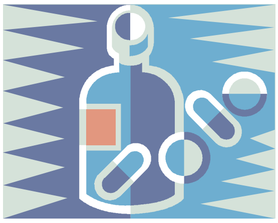

b) Om JA: Varför hämtade Du inte ut medicinen?

Du kan ange flera alternativ.

1 ☐ Jag blev frisk

1 ☐ Jag hade medicin så det räckte

1 ☐ Jag hade inte råd

1 ☐ Jag hade för lång väg till apoteket

1 ☐ Medicinen hjälper ändå inte

1 ☐ Annat ____________

Sömn och välbefinnande

Fråga15 Tycker Du att Du får tillräckligt med sömn för att känna Dig utvilad?

1 ☐ Ja, som regel
2 ☐ Ja, men inte tillräckligt ofta
3 ☐ Nej, aldrig eller nästan aldrig

Fråga 16 Har Du under de senaste 14 dagarna störts av sömnbesvär eller sömn-problem och hur besvärad har Du i så fall varit av dem?

1 ☐ Ja, mycket besvärad
2 ☐ Ja, lite besvärad
3 ☐ Nej

Fråga 17 Känner Du Dig stressad i Din vardag?

1 ☐ Ja, ofta
2 ☐ Ja, ibland
3 ☐ Nej, (nästan aldrig)

Fråga 18 Har Du under senaste månaden ofta känt Dig besvärad av att Du känt Dig nedstämd, deprimerad eller uppgiven?

1 ☐ Ja
2 ☐ Nej

Fråga 19 Har Du under senaste månaden ofta känt Dig besvärad av att Du känt Dig ointresserad eller haft mindre glädje än vanligt av att göra olika saker?

1 ☐ Ja
2 ☐ Nej

Fråga 20 Har Du de senaste veckorna kunnat koncentrera Dig på allt Du gjort?

1 ☐ Bättre än vanligt
2 ☐ Som vanligt
3 ☐ Sämre än vanligt
4 ☐ Mycket sämre än vanligt

Fråga 21 Har Du haft svårt att sova på grund av oro de senaste veckorna?

1 ☐ Inte alls
2 ☐ Inte mer än vanligt
3 ☐ Mer än vanligt
4 ☐ Mycket mer än vanligt

Fråga 22 Upplever Du att Du gjort nytta de senaste veckorna?

1 ☐ Mer än vanligt
2 ☐ Som vanligt
3 ☐ Mindre än vanligt
4 ☐ Mycket mindre än vanligt

Fråga 23 Har Du de senaste veckorna kunnat fatta beslut i olika frågor?

1 ☐ Bättre än vanligt
2 ☐ Som vanligt
3 ☐ Sämre än vanligt
4 ☐ Mycket sämre än vanligt

Fråga 24 Har Du ständigt känt Dig spänd de senaste veckorna?

1 ☐ Inte alls
2 ☐ Inte mer än vanligt
3 ☐ Mer än vanligt
4 ☐ Mycket mer än vanligt

Fråga 25 Har Du de senaste veckorna känt att Du inte kunnat klara Dina problem?

1 ☐ Inte alls
2 ☐ Inte mer än vanligt
3 ☐ Mer än vanligt
4 ☐ Mycket mer än vanligt

Fråga 26 Har Du de senaste veckorna känt att Du kunnat uppskatta det Du gjort om dagarna?

1 ☐ Mer än vanligt
2 ☐ Som vanligt
3 ☐ Mindre än vanligt
4 ☐ Mycket mindre än vanligt

Fråga 27 Har Du de senaste veckorna kunnat ta itu med Dina problem?

1 ☐ Bättre än vanligt
2 ☐ Som vanligt
3 ☐ Sämre än vanligt
4 ☐ Mycket sämre än vanligt

Fråga 28 Har Du de senaste veckorna känt Dig olycklig och nedstämd?

1 ☐ Inte alls
2 ☐ Inte mer än vanligt
3 ☐ Mer än vanligt
4 ☐ Mycket mer än vanligt

Fråga 29 Har Du de senaste veckorna förlorat tron på Dig själv?

1 ☐ Inte alls
2 ☐ Inte mer än vanligt
3 ☐ Mer än vanligt
4 ☐ Mycket mer än vanligt

Fråga 30 Har Du tyckt att Du varit värdelös de senaste veckorna?

1 ☐ Inte alls
2 ☐ Inte mer än vanligt
3 ☐ Mer än vanligt
4 ☐ Mycket mer än vanligt

Fråga 31 Har Du på det hela taget känt Dig någorlunda lycklig de senaste veckorna?

1 ☐ Mer än vanligt
2 ☐ Som vanligt
3 ☐ Mindre än vanligt
4 ☐ Mycket mindre än vanligt

Fråga 32 Hur känner Du Dig just nu, fysiskt och psykiskt, om Du ser till Din hälsa och Ditt välbefinnande?

Kryssa i lämplig ruta mellan 1 och 7 (1=mycket dåligt, 7=mycket bra)

	1	2	3	4	5	6	7	
Mycket dåligt<br>Kunde inte<br>må sämre	☐	☐	☐	☐	☐	☐	☐	Mycket bra<br>Kunde inte<br>må bättre

Tandhälsa

Fråga 33 När var Du senast hos tandläkare/ tandhygienist?

1 ☐ För mindre än 1 år sedan
2 ☐ För 1-2 år sedan
3 ☐ För 3-5 år sedan
4 ☐ För mer än fem år sedan
5 ☐ Har aldrig varit hos tandläkare/ tandhygienist

Fråga 34 Hur tycker Du att Din tandhälsa är?

1 ☐ Mycket bra
2 ☐ Ganska bra
3 ☐ Varken bra eller dålig
4 ☐ Ganska dålig
5 ☐ Mycket dålig

Fråga 35 Har Du under de senaste tre månaderna ansett Dig vara i behov av tandläkarvård, men inte sökt vård?

1 ☐ Nej ———> **Gå till fråga 37**
2 ☐ Ja

Fråga 36 Vilken var orsaken/orsakerna till att Du inte sökte vård?
Du kan ange flera alternativ.

1 ☐ Besvären gick över
1 ☐ Hade inte råd
1 ☐ Drar mig för att gå (tandläkarskräck)
1 ☐ Hade inte tid
1 ☐ Annan orsak. Vad? ____________

____

Fråga 37 Har Du något/några av följande besvär?

Sätt ett kryss på varje rad!	Nej<br>1	Ja, lätta besvär<br>2	Ja, svåra besvär<br>3
a) Besvär med karies (hål i tänderna)	☐	☐	☐
b) Blödande tandkött	☐	☐	☐
c) Tandlossning	☐	☐	☐
d) Tuggsvårigheter	☐	☐	☐
e) Känsliga tandhalsar	☐	☐	☐
f) Tandgnissling/pressning	☐	☐	☐

Fråga 38 Hur mycket har Du rört Dig och ansträngt Dig kroppsligt på fritiden under de senaste 12 månaderna?
Om Din aktivitet varierar mellan t.ex. sommar och vinter, försök att ta ett genomsnitt. Kryssa endast i ett alternativ!

1 ☐ Regelbunden motion och träning
Du ägnar Dig åt t.ex. löpning, simning, tennis, badminton, motionsgymnastik eller liknande vid i genomsnitt minst 3 tillfällen per vecka. Vardera tillfället varar minst 30 minuter per gång.

2 ☐ Måttlig, regelbunden motion på fritiden
Du motionerar regelbundet 1-2 gånger per vecka minst 30 minuter per gång med löpning, simning, tennis, badminton eller annan aktivitet som gör att Du svettas.

3 ☐ Måttlig motion på fritiden
Du promenerar, cyklar eller rör Dig på annat sätt under minst 2 timmar i veckan oftast utan att svettas. I detta inräknas också promenad eller cykling till och från arbetet, övriga promenader, ordinärt trädgårdsarbete, fiske, bordtennis, bowling.

4 ☐ Stillasittande fritid
Du ägnar Dig mest åt läsning, TV, bio eller annan stillasittande sysselsättning på fritiden. Du promenerar, cyklar eller rör Dig på annat sätt mindre än 2 timmar i veckan.

Fråga 39 Hur mycket tid ägnar Du en vanlig vecka åt måttligt ansträngande aktiviteter som får Dig att bli varm?
t.ex. promenader i rask takt, trädgårdsarbete, tyngre hushållsarbete, cykling, simning.

*Det kanske varierar under året, men försök ta något slags genomsnitt
Ange ett alternativ!*

1 ☐ 5 timmar per vecka eller mer
2 ☐ Mer än 3 timmar, men mindre än 5 timmar per vecka
3 ☐ Mellan 1 och 3 timmar per vecka
4 ☐ Högst en timme per vecka
5 ☐ Inte alls
6 ☐ Vet inte/kan inte ta ställning

Fråga 40 Vill Du öka Din fysiska aktivitet?

1 ☐ Ja, och jag tror att jag kan klara det själv
2 ☐ Ja, men jag behöver stöd
3 ☐ Nej

Fråga 41 Hur ofta äter Du grönsaker och rotfrukter?
Gäller alla typer av grönsaker, baljväxter och rotfrukter utom potatis (färska, frysta, konserverade, stuvade, juicer, soppor m.m.)

*Det kanske varierar under året, men försök ta något slags genomsnitt.
Ange ett alternativ!*

1 ☐ 3 gånger per dag eller oftare
2 ☐ 2 gånger per dag
3 ☐ 1 gång per dag
4 ☐ 5-6 gånger per vecka
5 ☐ 3-4 gånger per vecka
6 ☐ 1-2 gånger per vecka
7 ☐ Några gånger per månad eller aldrig

Fråga 42 Hur ofta äter Du frukt och bär?
Gäller alla typer av frukt och bär (färska, frysta, konserverade, juicer, kompott m.m.)

*Det kanske varierar under året, men försök ta något slags genomsnitt
Ange ett alternativ!*

1 ☐ 3 gånger per dag eller oftare
2 ☐ 2 gånger per dag
3 ☐ 1 gång per dag
4 ☐ 5-6 gånger per vecka
5 ☐ 3-4 gånger per vecka
6 ☐ 1-2 gånger per vecka
7 ☐ Några gånger per månad eller aldrig

Fråga 43 Vilken typ av matfett brukar Du vanligtvis använda på smörgås?
Markera ett alternativ, det vanligaste!

1 ☐ Smör/Bordsmargarin 80% fett, t.ex. Bregott, Flora, Linnéa
2 ☐ Hushållsmargarin (margarin i folie), t.ex. Milda, HushållsEve, Ädel
3 ☐ Mellanmargarin 60% fett, t.ex. Bregott Mellan, Runda Bords
4 ☐ Lättmargarin 30-40% fett, t.ex. Lätta, Lätt&Lagom, Becel, LättLätt, Gaio,
5 ☐ Använder ej matfett på smörgås/Äter ej smörgås

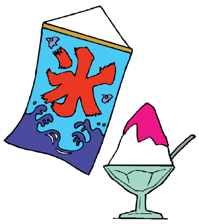

Fråga 44 När Du väljer vad Du ska äta, hur viktigt är det då att maten är "näringsriktig" eller "hälsosam"?

1 ☐ Det är mycket viktigt
2 ☐ Det är ganska viktigt
3 ☐ Det är oviktigt
4 ☐ Vet inte/har ingen åsikt

Fråga 45 Har Du under det senaste året ändrat eller försökt ändra Dina matvanor för att äta mer hälsosamt?

1 ☐ Ja, jag har börjat äta mer hälsosamt
2 ☐ Ja, jag har försökt, men misslyckats
3 ☐ Nej, men jag har funderat på det
4 ☐ Nej, aldrig
5 ☐ Nej, jag anser att jag redan äter hälsosamt
6 ☐ Jag har redan ändrat matvanorna sedan tidigare

Fråga 46 a) Röker Du?

1 ☐ Ja, dagligen
2 ☐ Ja, men inte alla dagar
3 ☐ Nej

(2–3) **Gå till fråga 47**

b) Om JA DAGLIGEN: Hur mycket röker Du i genomsnitt?

☐☐☐ cigaretter dagligen
☐☐☐ cigariller dagligen
☐☐☐ cigarrer dagligen
☐☐☐ gram piptobak i veckan

Gå till fråga 48

Fråga 47 a) Har Du tidigare rökt dagligen under <u>minst 6 månader</u>?

1 ☐ Nej ———> **Gå till fråga 49**
2 ☐ Ja

b) Om JA: För hur länge sedan slutade Du röka dagligen?

1 ☐ Mindre än 6 månader sedan
2 ☐ Mellan 6 och 12 månader sedan
3 ☐ Mer än 1 år sedan,

ange vilket år ☐☐☐☐

c) Hur slutade Du röka?

1 ☐ På egen hand utan nikotinersättningsmedel
2 ☐ På egen hand med hjälp av nikotinersättningsmedel
3 ☐ Har fått professionell hjälp
4 ☐ Började snusa i stället

Fråga 48 Hur gammal var Du när Du började röka dagligen?

☐☐ år

Fråga 49 Vill Du sluta röka?

1 ☐ Röker ej
2 ☐ Ja, och jag tror att jag kan klara det själv
3 ☐ Ja, men jag behöver stöd
4 ☐ Nej

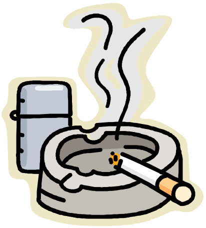

Fråga 50 Hur ofta vistas Du i lokaler där andra personer röker eller nyss har rökt?

Sätt ett kryss på varje rad!	Varje dag<br>1	Någon/några gånger i veckan<br>2	Någon/några gånger i månaden<br>3	Mer sällan eller aldrig<br>4
a) I Ditt hem	☐	☐	☐	☐
b) På arbetet	☐	☐	☐	☐
c) På café, bar eller restaurang	☐	☐	☐	☐
d) På annan plats inomhus, t.ex. hos vänner, i bilen osv.	☐	☐	☐	☐

Fråga 51 Snusar Du dagligen?

1 ☐ Nej
2 ☐ Ja ———> **Gå till fråga 54**

Fråga 52 Händer det att Du snusar någon gång då och då?

1 ☐ Nej
2 ☐ Ja

Fråga 53 a) Har Du tidigare snusat dagligen under <u>minst 6 månader</u>?

1 ☐ Nej ———> **Gå till fråga 55**
2 ☐ Ja

b) Om JA: För hur länge sedan slutade Du snusa dagligen?

1 ☐ Mindre än 6 månader sedan
2 ☐ Mellan 6 och 12 månader sedan
3 ☐ Mer än 1 år sedan,

ange vilket år ☐☐☐☐

Fråga 54 Hur gammal var Du när Du började snusa dagligen?

☐☐ år

Fråga 55 Vill Du sluta snusa?

1 ☐ Snusar ej
2 ☐ Ja, och jag tror att jag kan klara det själv
3 ☐ Ja, men jag behöver stöd
4 ☐ Nej

Här kommer några frågor om Dina alkoholvanor. Vi är tacksamma om Du besvarar dem så noggrant och ärligt som möjligt genom att markera det alternativ som gäller för Dig. Med "alkohol" menas folköl, mellan-/starköl, alkohol-stark cider, vin, starkvin och sprit.

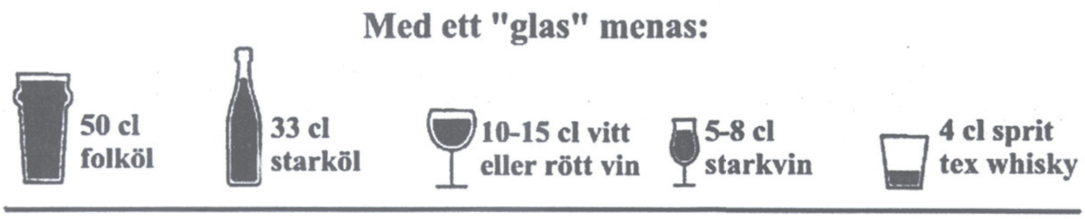

Fråga 56 Hur ofta har Du druckit alkohol under de senaste 12 månaderna?

1 ☐ 4 gånger/vecka eller mer
2 ☐ 2-3 gånger/vecka
3 ☐ 2-4 gånger/månad
4 ☐ 1 gång/månad eller mer sällan
5 ☐ Aldrig ———> **Gå till fråga 61**

Fråga 57 Hur många "glas" (se exempel) dricker Du en typisk dag då Du dricker alko-hol?

1 ☐ 1-2
2 ☐ 3-4
3 ☐ 5-6
4 ☐ 7-9
5 ☐ 10 eller fler

Fråga 58 Hur ofta dricker Du sex "glas" eller fler vid samma tillfälle?

1 ☐ Dagligen eller nästan varje dag
2 ☐ Varje vecka
3 ☐ Varje månad
4 ☐ Mer sällan än en gång i månaden
5 ☐ Aldrig

Fråga 59 Hur ofta under de senaste 12 måna-derna har Du druckit så mycket alkohol att Du varit berusad?

1 ☐ Dagligen eller nästan dagligen
2 ☐ Några gånger/vecka
3 ☐ 1 gång/vecka
4 ☐ 2-3 gånger/månad
5 ☐ 1 gång/månad
6 ☐ Någon eller några gånger per halvår
7 ☐ Mer sällan eller aldrig

Fråga 60 **Vill Du minska Din alkoholkonsumtion?**

1 ☐ Ja, och jag tror att jag kan klara det själv
2 ☐ Ja, men jag behöver stöd
3 ☐ Nej

Fråga 61 **Har Du under de senaste 12 månaderna tagit med Dig alkohol hem till Sverige när Du kom hem från en utlandsresa?**

1 ☐ Ej varit utomlands de senaste 12 månaderna
2 ☐ Ja
3 ☐ Nej

Fråga 62 **Har Du under de senaste 12 månaderna inköpt märkessprit av en privatperson till ett lägre pris än vad som gäller i Systembolagets butiker?**

Med märkessprit menas flaska med etikett från officiell spritttillverkare.

1 ☐ Ja, regelbundet
2 ☐ Ja, flera gånger
3 ☐ Ja, någon gång
4 ☐ Nej, aldrig

Fråga 63 **Har Du någonsin rökt hasch eller marijuana?**

1 ☐ Nej
2 ☐ Ja, inom den senaste månaden
3 ☐ Ja, inom det senaste året
4 ☐ Ja, för mer än ett år sedan

Fråga 64 **Har Du någon gång under de senaste 12 månaderna köpt lotter eller satsat pengar på spel?**

Med spel menas t.ex. trisslott, bingolotto, kasinospel, tips, spel på hästar eller liknande.

1 ☐ Nej ———> **Gå till fråga 67**
2 ☐ Ja

Fråga 65 **Hur mycket pengar har Du satsat på spel de senaste 7 dagarna?**

Har spelat för ☐☐☐☐☐☐ kronor

☐ Har inte satsat några pengar de senaste 7 dagarna

Fråga 66 **Hur många gånger har Du under de senaste 12 månaderna ...**

Sätt ett kryss på varje rad!	Aldrig<br>1	1-2 gånger<br>2	3 gånger eller fler<br>3
a) försökt minska Ditt spelande?	☐	☐	☐
b) känt Dig rastlös och irriterad om Du inte kunnat spela?	☐	☐	☐
c) ljugit om hur mycket Du spelat?	☐	☐	☐

Sociala relationer

Fråga 67 **Känner Du att Du har någon eller några personer som kan ge Dig ett ordentligt personligt stöd för att klara av livets stress och problem?**

1 ☐ Ja, helt säkert
2 ☐ Ja, troligen
3 ☐ Inte helt säkert
4 ☐ Nej

Fråga 68 **Kan Du få hjälp av någon eller några personer vid sjukdom eller praktiska problem (låna småsaker, hjälp till reparation, hjälp att skriva en skrivelse, få råd eller information)?**

1 ☐ Ja, utan tvekan
2 ☐ Ja, troligen
3 ☐ Nej, troligen inte
4 ☐ Nej, inte alls

Fråga 69 Har Du under de senaste 12 månaderna ...

Kryss kan sättas i flera rutor!

1 ☐ deltagit i studiecirkel/kurs på Din arbetsplats
1 ☐ deltagit i studiecirkel/kurs på Din fritid
1 ☐ deltagit i fackföreningsmöte
1 ☐ deltagit i annat föreningsmöte
1 ☐ varit på teater/bio
1 ☐ varit på konstutställning
1 ☐ deltagit i religiös sammankomst
1 ☐ varit på sportevenemang
1 ☐ skrivit insändare i tidning/tidskrift
1 ☐ deltagit i demonstration av något slag
1 ☐ besökt offentlig tillställning, exempelvis nattklubb, danstillställning eller liknande
1 ☐ deltagit i större släktsammankomst
1 ☐ varit på privat fest hos någon
1 ☐ inget av ovanstående

Fråga 70 Är Du rotad och känner en stark samhörighet med Ditt bostadsområde?

1 ☐ I hög grad
2 ☐ I viss mån
3 ☐ Inte speciellt
4 ☐ Inte alls

Fråga 71 Är Du rotad och känner en stark samhörighet med Dina arbetskamrater?

1 ☐ Arbetar ej
2 ☐ Har inga arbetskamrater
3 ☐ I hög grad
4 ☐ I viss mån
5 ☐ Inte speciellt
6 ☐ Inte alls

Fråga 72 Ta ställning till följande påståenden:

a) De flesta människor skulle utnyttja Dig om de fick chansen.

1 ☐ Håller inte alls med
2 ☐ Håller inte med
3 ☐ Håller med
4 ☐ Håller med fullständigt

b) De flesta människor försöker vara i grunden rättvisa.

1 ☐ Håller inte alls med
2 ☐ Håller inte med
3 ☐ Håller med
4 ☐ Håller med fullständigt

c) Man kan lita på de flesta människor.

1 ☐ Håller inte alls med
2 ☐ Håller inte med
3 ☐ Håller med
4 ☐ Håller med fullständigt

d) Man kan inte vara nog försiktig när man har med andra människor att göra.

1 ☐ Håller inte alls med
2 ☐ Håller inte med
3 ☐ Håller med
4 ☐ Håller med fullständigt

Fråga 73 Har Du under de senaste 12 månaderna blivit personligen angripen med anledning av Din ras eller hudfärg?

1 ☐ Ja, genom att någon sagt eller skrikit något till Dig
1 ☐ Ja, genom att någon fysiskt angripit Din person eller förstört eller vandaliserat något som tillhörde Dig
1 ☐ Ja, på annat sätt
1 ☐ Nej

Fråga 74 a) Tror Du att det finns arbetsgivare i Sverige som skulle vägra att ge ett arbete till en person på grund av hans eller hennes ras, hudfärg, religiösa tillhörighet eller kulturella bakgrund?

1 ☐ Inga eller väldigt få
2 ☐ Mindre än hälften
3 ☐ Ungefär hälften
4 ☐ De flesta

b) Vilka av följande alternativ anser Du vara bland de viktigaste målen för svensk politik? (Välj max två alternativ):

1 ☐ Upprätthålla lag och ordning i landet
1 ☐ Ge folk mer inflytande över politiska beslut
1 ☐ Bekämpa ökande priser
1 ☐ Försvara yttrandefriheten
1 ☐ Vet ej/ingen åsikt

Hot om våld

Fråga 75 Har Du någon gång under de senaste tolv månaderna blivit utsatt för hot eller hotelser om våld som var så farliga eller så allvarliga att Du blev rädd?

1 ☐ Ja
2 ☐ Nej

Fråga 76 a) Har Du någon gång under de senaste tolv månaderna blivit utsatt för fysiskt våld?

1 ☐ Ja
2 ☐ Nej ———> **Gå till fråga 77**

b) Om JA: Var skedde detta?
Du får kryssa flera alternativ!

1 ☐ På arbetsplatsen/i arbetet /skolan
1 ☐ I hemmet
1 ☐ I annans bostad/i bostadsområdet
1 ☐ På allmän plats/på nöjesställe/på tåg, buss, tunnelbana
1 ☐ Någon annanstans

c) Om JA: Ledde detta våld till sådan kroppsskada att Du måste söka läkar-vård?

1 ☐ Ja
2 ☐ Nej

Förtroende för samhällsinstitutioner

Fråga 77 Vilket förtroende har Du för följande institutioner i samhället?

Sätt ett kryss på varje rad!	Mycket stort<br>1	Ganska stort<br>2	Inte särskilt stort<br>3	Inget alls<br>4	Har ingen åsikt<br>5
a) Sjukvården	☐	☐	☐	☐	☐
b) Skolan	☐	☐	☐	☐	☐
c) Polisen	☐	☐	☐	☐	☐
d) Socialtjänsten	☐	☐	☐	☐	☐
e) Arbetsförmedlingen	☐	☐	☐	☐	☐
f) Försäkringskassan	☐	☐	☐	☐	☐
g) Domstolar	☐	☐	☐	☐	☐
h) Riksdagen	☐	☐	☐	☐	☐
i) Politiker i Ditt landsting	☐	☐	☐	☐	☐
j) Politiker i kommunen	☐	☐	☐	☐	☐
k) Massmedia, TV, tidningar	☐	☐	☐	☐	☐

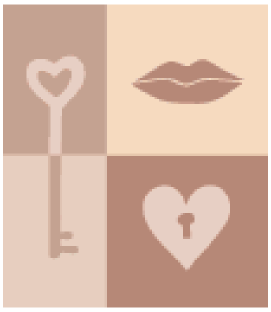

Arbete, sysselsättning och ekonomi

Fråga 78 a) Vilket är/var Ditt yrke eller Dina arbetsuppgifter?

Om Du inte förvärvsarbetar nu, ange yrke/arbetsuppgifter som Du huvudsakligen har haft.
Om du aldrig förvärvsarbetat gå till fråga 79.

Försök att lämna en yrkesbeskrivning som noggrannt beskriver Din huvudsakliga arbetsuppgift. I stället för chaufför, skriv t.ex. busschaufför, lastbilschaufför etc.

Exempel: I stället för lärare, skriv t.ex.

F	Ö	R	S	K	O	L	E	L	Ä	R	A	R	E								

Yrke:

Beskriv även dina huvudsakliga arbetsuppgifter. Om Du är t.ex. projektledare skriv då t.ex. ansvarig för att förbättra arbetsmiljön i åldringsvården, ansvarig för att utveckla systemet för att förkorta väntetider i call center-verksamhet.

b) Beskriv dina huvudsakliga arbetsuppgifter så noggrant som möjligt.

Fråga 79 Vilken är Din nuvarande sysselsättning?

Du kan ange flera alternativ!

1 ☐ Yrkesarbetar ☐☐☐ % av heltid
1 ☐ Tjänstledig eller föräldraledig
1 ☐ Studerar, praktiserar
1 ☐ Arbetsmarknadsåtgärd
1 ☐ Arbetslös
1 ☐ Ålderspensionär
1 ☐ Avtalspensionär
1 ☐ Förtidspensionerad, sjukpensionerad
1 ☐ Långtidssjukskriven (mer än 3 månader)
1 ☐ Sköter eget hushåll
1 ☐ Annat. Vad? ____

Nedan följer några frågor till Dig som förvärvsarbetar (även Du som är sjukskriven eller föräldra-/ tjänstledig). Övriga går vidare till fråga 96.

Fråga 80 Vilken är Din huvudsakliga arbetsgivare? *Ange ett alternativ!*

1 ☐ Privatägt företag
2 ☐ Statlig myndighet/Statligt ägt bolag eller verk
3 ☐ Region/Landsting/Landstingsägt bolag
4 ☐ Kommun/Kommunägt bolag
5 ☐ Driver eget företag
6 ☐ Annat. Vad? ____

Fråga 81 a) Har Du förvärvsarbetat under de senaste 12 månaderna?

1 ☐ Nej
2 ☐ Ja

b) Om JA: Hur många månader har Du förvärvsarbetat under de senaste 12 månaderna?

☐☐ månader

Fråga 82 Vilken är Din nuvarande anställningsform?
Ange ett alternativ!

1 ☐ Egen företagare
2 ☐ Fast anställd (tillsvidareanställd)
3 ☐ Vikarieanställd
4 ☐ Projektanställd
5 ☐ Timanställd
6 ☐ Anställd på personaluthyrnings- eller bemanningsföretag
7 ☐ Provanställd
8 ☐ Annan anställning

Fråga 83 Om du är anställd eller egen företagare, vilket är ditt anställningsförhållande?

1 ☐ Anställd
2 ☐ Egen företagare utan anställda
3 ☐ Egen företagare med 1-9 anställda
4 ☐ Egen företagare med 10 eller fler anställda

Fråga 84 a) Har Du varit ofrivilligt arbetslös någon gång under de senaste tre åren?

1 ☐ Ja
2 ☐ Nej

b) Om JA: Ungefär hur lång tid har Du sammanlagt varit arbetslös under de senaste tre åren?

☐☐ månader

Fråga 85 Är Du orolig för att förlora Ditt arbete inom det närmaste året?

1 ☐ Inte alls orolig
2 ☐ Inte särskilt orolig
3 ☐ Ganska orolig
4 ☐ Mycket orolig

Fråga 86 Det företag/den arbetsplats Du idag arbetar på, är det den arbetsplats Du önskar arbeta på i framtiden?

1 ☐ Ja
2 ☐ Nej

Fråga 87 Är det yrke Du har idag det yrke Du önskar ha i framtiden?

1 ☐ Ja
2 ☐ Nej

Fråga 88 Kan Du anpassa Ditt arbete om Du känner Dig hängig, har värk, är förkyld eller liknande?

1 ☐ Sällan eller aldrig
2 ☐ Ibland
3 ☐ Ofta

Fråga 89 Kan det på grund av arbetet vara svårt för Dig att stanna hemma om Du är sjuk en eller två dagar?

1 ☐ Sällan eller aldrig
2 ☐ Ibland
3 ☐ Ofta

Fråga 90 Hur många gånger under de senaste 12 månaderna har det hänt att Du gått till arbetet trots att Du med tanke på Ditt hälsotillstånd egentligen borde ha sjukskrivit Dig?

1 ☐ Ingen gång
2 ☐ En gång
3 ☐ Några gånger
4 ☐ Många gånger

Arbetsmiljö

Om Du inte förvärvsarbetat alls under de senaste 12 månaderna gå till fråga 96.

Fråga 91 Håller Du med om följande påståenden?

a) Mitt arbete kräver att jag lär mig nya saker

1 ☐ Håller inte alls med
2 ☐ Håller inte med
3 ☐ Håller med
4 ☐ Håller med fullständigt

b) Mitt arbete innebär att jag gör samma sak om och om igen

1 ☐ Håller inte alls med
2 ☐ Håller inte med
3 ☐ Håller med
4 ☐ Håller med fullständigt

c) I mitt arbete måste man vara påhittig och kreativ

1 ☐ Håller inte alls med
2 ☐ Håller inte med
3 ☐ Håller med
4 ☐ Håller med fullständigt

d) I mitt arbete får jag fatta egna beslut

1 ☐ Håller inte alls med
2 ☐ Håller inte med
3 ☐ Håller med
4 ☐ Håller med fullständigt

e) Mitt arbete kräver stor skicklighet

1 ☐ Håller inte alls med
2 ☐ Håller inte med
3 ☐ Håller med
4 ☐ Håller med fullständigt

f) Jag har mycket lite frihet att bestämma hur mitt arbete ska utföras

1 ☐ Håller inte alls med
2 ☐ Håller inte med
3 ☐ Håller med
4 ☐ Håller med fullständigt

g) Jag får göra många olika saker i mitt arbete

1 ☐ Håller inte alls med
2 ☐ Håller inte med
3 ☐ Håller med
4 ☐ Håller med fullständigt

h) Jag har mycket att säga till om vad det gäller det som händer på mitt arbete

1 ☐ Håller inte alls med
2 ☐ Håller inte med
3 ☐ Håller med
4 ☐ Håller med fullständigt

i) Jag har möjlighet att utveckla min egen förmåga

1 ☐ Håller inte alls med
2 ☐ Håller inte med
3 ☐ Håller med
4 ☐ Håller med fullständigt

j) Mitt arbete kräver att jag arbetar mycket snabbt

1 ☐ Håller inte alls med
2 ☐ Håller inte med
3 ☐ Håller med
4 ☐ Håller med fullständigt

k) Mitt arbete kräver att jag arbetar mycket hårt

1 ☐ Håller inte alls med
2 ☐ Håller inte med
3 ☐ Håller med
4 ☐ Håller med fullständigt

l) Man kräver inte för stor arbetsinsats av mig

1 ☐ Håller inte alls med
2 ☐ Håller inte med
3 ☐ Håller med
4 ☐ Håller med fullständigt

m) Jag har tillräckligt med tid för att få arbetet utfört

1 ☐ Håller inte alls med
2 ☐ Håller inte med
3 ☐ Håller med
4 ☐ Håller med fullständigt

n) Jag slipper motstridiga krav från andra

1 ☐ Håller inte alls med
2 ☐ Håller inte med
3 ☐ Håller med
4 ☐ Håller med fullständigt

o) I mitt arbete krävs långa perioder av intensiv koncentration på uppgiften

1 ☐ Håller inte alls med
2 ☐ Håller inte med
3 ☐ Håller med
4 ☐ Håller med fullständigt

p) Jag avbryts ofta i mina arbetsuppgifter innan de är färdiga och måste senare ta itu med dem

1 ☐ Håller inte alls med
2 ☐ Håller inte med
3 ☐ Håller med
4 ☐ Håller med fullständigt

q) Mitt arbete är mycket hektiskt (stressigt)

1 ☐ Håller inte alls med
2 ☐ Håller inte med
3 ☐ Håller med
4 ☐ Håller med fullständigt

r) Jag måste ofta slå av på takten i mitt arbete för att vänta på att arbetskamrater eller andra avdelningar ska bli klara med sina uppgifter

1 ☐ Håller inte alls med
2 ☐ Håller inte med
3 ☐ Håller med
4 ☐ Håller med fullständigt

s) Min arbetsledare (chef) bryr sig om dem som arbetar under honom

1 ☐ Saknar/saknade arbetsledare
2 ☐ Håller inte alls med
3 ☐ Håller inte med
4 ☐ Håller med
5 ☐ Håller med fullständigt

t) Min arbetsledare (chef) lyssnar på vad jag har att säga

1 ☐ Saknar/saknade arbetsledare
2 ☐ Håller inte alls med
3 ☐ Håller inte med
4 ☐ Håller med
5 ☐ Håller med fullständigt

u) Min arbetsledare (chef) hjälper mig att få jobbet gjort

1 ☐ Saknar/saknade arbetsledare
2 ☐ Håller inte alls med
3 ☐ Håller inte med
4 ☐ Håller med
5 ☐ Håller med fullständigt

v) Min arbetsledare (chef) är bra på att få folk att samarbeta

1 ☐ Saknar/saknade arbetsledare
2 ☐ Håller inte alls med
3 ☐ Håller inte med
4 ☐ Håller med
5 ☐ Håller med fullständigt

w) Mina arbetskamrater är duktiga i sina jobb

1 ☐ Saknar/saknade arbetskamrater
2 ☐ Håller inte alls med
3 ☐ Håller inte med
4 ☐ Håller med
5 ☐ Håller med fullständigt

x) Mina arbetskamrater är intresserade av mig som människa

1 ☐ Saknar/saknade arbetskamrater
2 ☐ Håller inte alls med
3 ☐ Håller inte med
4 ☐ Håller med
5 ☐ Håller med fullständigt

y) Mina arbetskamrater är vänliga

1 ☐ Saknar/saknade arbetskamrater
2 ☐ Håller inte alls med
3 ☐ Håller inte med
4 ☐ Håller med
5 ☐ Håller med fullständigt

z) Mina arbetskamrater hjälper till att få jobbet gjort

1 ☐ Saknar/saknade arbetskamrater
2 ☐ Håller inte alls med
3 ☐ Håller inte med
4 ☐ Håller med
5 ☐ Håller med fullständigt

Fråga 92 a) Händer det att Du arbetar övertid på Ditt arbete?

1 ☐ Arbetar aldrig övertid
2 ☐ Arbetar övertid någon gång i månaden
3 ☐ Arbetar ofta övertid
4 ☐ Har ej reglerad arbetstid

b) Om Du ARBETAR ÖVERTID: Ange genomsnittligt antal övertidstimmar per vecka

☐☐ timmar per vecka

c) Om Du ARBETAR ÖVERTID: Kan Du vanligen själv bestämma när Du vill förlägga övertiden?

1 ☐ Ja
2 ☐ Nej

d) Om Du ARBETAR ÖVERTID: Får Du någon form av övertidsersättning?

1 ☐ Ja
2 ☐ Nej

Fråga 93 Hur ofta förekommer följande inslag i Ditt arbete?
Sätt ett kryss på varje rad!

	Varje dag 1	Några dagar i veckan 2	Mera sällan 3	Aldrig 4
a) Buller (måste höja rösten vid samtal)	☐	☐	☐	☐
b) Kemiska preparat, ångor, gaser	☐	☐	☐	☐
c) Upprepade och ensidiga arbetsrörelser	☐	☐	☐	☐
d) Tunga lyft (mer än 20 kg/lyft)	☐	☐	☐	☐
e) Mobbning eller trakasserier	☐	☐	☐	☐

Fr 94 a) Erbjuder Din arbetsgivare någon form av ersättning för motion eller träning?

Du kan ange flera alternativ!

1 ☐ Nej, sådana möjligheter finns inte
1 ☐ Ej aktuellt
1 ☐ Möjlighet att träna/motionera på arbetstid, t.ex. friskvårdstimme
1 ☐ Subventionerat träningskort, gratis simning eller liknande
1 ☐ Andra möjligheter till träning,
vad? ______________________

____

Fr 94 b) Utnyttjar Du denna/dessa förmåner?

1 ☐ Ja, ofta
2 ☐ Ja,ibland
3 ☐ Nej, aldrig

Fr 95 a) Hur tar Du Dig vanligtvis till arbetet?
Flera alternativ är möjliga.

1 ☐ Promenad
1 ☐ Cykel
1 ☐ Bil
1 ☐ Buss
1 ☐ Tåg
1 ☐ Annat

Fr 95 b) Hur lång tid tar det att komma till arbetet (enkel resa)?

1 ☐ Mindre än 15 minuter
2 ☐ 15-30 minuter
3 ☐ 30-60 minuter
4 ☐ 1-1,5 timme
5 ☐ 1,5-2 timmar
6 ☐ Mer än 2 timmar

Fråga 96 I vilken utsträckning tror du att följande faktorer påverkar sjukskrivningarna i Sverige?

	Mycket stor 1	Ganska stor 2	Inte särskilt stor 3	Ingen 4	Har ingen åsikt 5
a) Påfrestande arbetsförhållanden	☐	☐	☐	☐	☐
b) Påfrestningar utanför arbetet	☐	☐	☐	☐	☐
c) Ersättningsnivåerna vid sjukskrivning	☐	☐	☐	☐	☐
d) Läkarnas sjukskrivningsvanor	☐	☐	☐	☐	☐
e) Försäkringskassans arbetssätt	☐	☐	☐	☐	☐

Fråga 97 Vilken betydelse anser Du att följande faktorer kan ha för Din egen risk att bli sjukskriven?

	Mycket stor 1	Ganska stor 2	Inte särskilt stor 3	Ingen 4	Ingen åsikt/ ej aktuellt 5
a) En dålig arbetsmiljö på grund skadliga ämnen, buller, belastande arbetsställningar/lyft	☐	☐	☐	☐	☐
b) För stora psykologiska krav i arbetet	☐	☐	☐	☐	☐
c) För lite inflytande över arbetssituationen	☐	☐	☐	☐	☐
d) Dåligt stöd från arbetsledarna	☐	☐	☐	☐	☐
e) Dålig stämning bland arbetskamraterna	☐	☐	☐	☐	☐
f) Oro för att förlora jobbet	☐	☐	☐	☐	☐
g) Problem i familjen/privatlivet	☐	☐	☐	☐	☐
h) Svårt att få tid för behandling av hälsoproblem	☐	☐	☐	☐	☐

Hem, hushåll och boendemiljö

Fråga 98 Vilken typ av bostad bor Du i?

1 ☐ Egen villa/radhus
2 ☐ Bostadsrätt/andelsrätt
3 ☐ Hyresrätt
4 ☐ Inneboende, studentlägenhet/rum
5 ☐ Annat

Fråga 99 Hur stor är bostadsytan?
Uppskatta om Du inte vet exakt.

☐☐☐ kvadratmeter

Fråga 100 Hur många personer bor i familjens bostad?

☐☐ person/personer

Fråga 101 Hur många rum finns i bostaden? *(förutom köket)*

☐☐ rum

Fråga 102 Hur länge har Du bott i Din nuvarande bostad?
Ange antal år.

☐☐ år

Fråga 103 Med vem delar Du bostad?
Dvs. bor tillsammans med under större delen av veckan. Du kan ange flera alternativ.

1 ☐ Ingen
1 ☐ Föräldrar/Syskon
1 ☐ Make/Maka/Sambo/Partner
1 ☐ Andra vuxna
1 ☐ Barn ——> Hur gamla är de barn som bor hemma?

1 ☐ 0-6 år
1 ☐ 7-12 år
1 ☐ 13-17 år
1 ☐ 18 år eller äldre

Fråga 104 Hur många timmar per vecka lägger Du ned på arbete i hemmet (som inte är yrkesarbete)?

T.ex. handla, laga mat, sköta ekonomin, tvätta, städa, sköta barn, underhålla bil, hus, trädgård.

1 ☐ 0-2 timmar/vecka
2 ☐ 3-10 timmar/vecka
3 ☐ 11-20 timmar/vecka
4 ☐ 21-30 timmar/vecka
5 ☐ 31 timmar/vecka eller mer

Fråga 105 a) Har Du någon gammal, sjuk eller funktionshindrad närstående som Du behöver hjälpa med vardagliga sysslor, se till eller vårda?

1 ☐ Nej ——> **Gå till fråga 106**
2 ☐ Ja

b) Om JA: Hur många timmars bundenhet /arbete per vecka innebär detta för Dig?

☐☐ timmar per vecka

Fråga 106 Finns det något grönområde (större park eller liknande) eller skogsområde inom 5-10 minuteras gångavstånd från där Du bor?

1 ☐ Ja
2 ☐ Nej
3 ☐ Vet ej

Fråga 107 Hur säker och trygg känner Du Dig när Du går ensam i Ditt bostadsområde när det är mörkt?

1 ☐ Mycket säker
2 ☐ Ganska säker
3 ☐ Ganska osäker
4 ☐ Mycket osäker
5 ☐ Är aldrig ute ensam när det är mörkt

Fråga 108 Hur trivs Du i området där Du bor?

1 ☐ Mycket bra
2 ☐ Ganska bra
3 ☐ Ganska dåligt
4 ☐ Mycket dåligt
5 ☐ Vej ej/Ej aktuellt

Fråga 109 Tänk på omgivningen där Du bor, tycker Du att ...

Sätt kryss på varje rad!	Ja<br>1	Nej<br>2	Ingen åsikt<br>3
a) servicen är bra? *t.ex. närhet till butiker, bank, post, barnomsorg*	☐	☐	☐
b) kulturutbudet är bra? *t.ex. närhet till bibliotek, bio, teater*	☐	☐	☐
c) fritidsmöjligheterna är bra? *t.ex. närhet till simhall, idrottsanläggningar, motionsspår*	☐	☐	☐
d) de allmänna kommunikationerna är tillfredsställande?	☐	☐	☐
e) det finns störande ljud? *t.ex. trafik/flygbuller, industrier, krogar, grannar, fläktar*	☐	☐	☐
f) det finns besvärande luftföroreningar? *t.ex. avgaser, lukt, damm, sot*	☐	☐	☐

Fråga 110 Har du de senaste 3 månaderna känt Dig besvärad av något av följande i eller i närheten av Din bostad?

	Ja, minst en gång per dag<br>1	Ja, minst en gång per vecka<br>2	Ja, mer sällan<br>3	Nej, aldrig<br>4
a) Ljud från grannar	☐	☐	☐	☐
b) Vägtrafikbuller	☐	☐	☐	☐
c) Tågbuller	☐	☐	☐	☐
d) Flygbuller	☐	☐	☐	☐
e) Bilavgaser	☐	☐	☐	☐
e) Vedeldningsrök	☐	☐	☐	☐
e) Lukt från industrier	☐	☐	☐	☐

Fråga 111 Medför trafikbuller (väg-, tåg- eller flygtrafik) några av följande störningar i din bostad?

	Ja, minst en gång per dag 1	Ja, minst en gång per vecka 2	Ja, mer sällan 3	Nej, aldrig 4
a) Svårt att höra radio/TV	☐	☐	☐	☐
b) Telefonsamtal hindras	☐	☐	☐	☐
c) Vanligt samtal hindras	☐	☐	☐	☐
d) Vila/avkoppling störs	☐	☐	☐	☐
e) Svårt att somna	☐	☐	☐	☐
f) Bli väckt	☐	☐	☐	☐

Bakgrundsdata

Fråga 112 Vilket år är Du född?

19☐☐

Fråga 113 Är du man eller kvinna?

1 ☐ Man
2 ☐ Kvinna

Fråga 114 Vilket är ditt civilstånd?

1 ☐ Gift/sambo
2 ☐ Ogift
3 ☐ Frånskild
4 ☐ Änka/änkling

Fråga 115 a) Är Du född i Sverige?

1 ☐ Ja ——> **Gå till fråga 116**
2 ☐ Nej

b) Om NEJ: Vilket land är Du född i?

☐☐☐☐☐☐☐☐☐☐☐☐☐☐☐☐☐☐☐☐☐☐

c) Om NEJ: När flyttade du till Sverige?

År: ☐☐☐☐

Fråga 116 a) Är Dina föräldrar födda i Sverige?

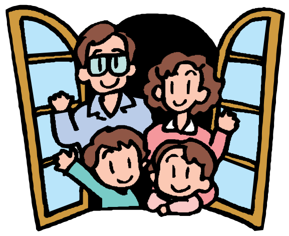

1 ☐ Ja, båda ——> **Gå till fråga 117**
2 ☐ Nej, en av föräldrarna är född i annat land
3 ☐ Båda föräldrarna är födda i annat land

b) OM NÅGON ÄR FÖDD UTOMLANDS: I vilket eller vilka länder?

Faderns födelseland:

☐☐☐☐☐☐☐☐☐☐☐☐☐☐☐☐☐☐☐☐☐☐

Moderns födelseland:

☐☐☐☐☐☐☐☐☐☐☐☐☐☐☐☐☐☐☐☐☐☐

Fråga 117 Vilket språk används huvudsakligen i Ditt hushåll?

1 ☐ Svenska
2 ☐ Annat
3 ☐ Svenska och annat lika mycket

Fråga 118 Vilken är den högsta utbildningen Du har?

Om Du studerar kryssa i den utbildning Du går.

Sätt bara ett kryss!

1 ☐ Folkskola eller grundskola
2 ☐ Realskola eller flickskola
3 ☐ 2-årigt gymnasium eller yrkesskola
4 ☐ 3-4-årigt gymnasium
5 ☐ Universitet eller högskola, mindre än 3 år (mindre än 100 p)
6 ☐ Universitet eller högskola, 3 år eller längre (120 p eller mer)
7 ☐ Annan utbildning. Vilken?

Fråga 119 Hur ofta har det hänt att Du under de senaste 12 månaderna haft svårigheter att klara av Dina räkningar (hyra, el, tel, räntor, amorteringar, försäkringar etc.)?

1 ☐ Varje månad
2 ☐ Ungefär hälften av årets månader
3 ☐ Någon enstaka gång
4 ☐ Aldrig

Fråga 120 Om Du plötsligt skulle hamna i en oförutsedd situation, där Du på en vecka måste skaffa fram 14 000 kronor, skulle Du klara det?

1 ☐ Ja
2 ☐ Nej

Fråga 121 Hade Din familj det svårt ekonomiskt under Din uppväxttid?

1 ☐ Nej, inga nämnvärda ekonomiska problem
2 ☐ Ja, lätta och/eller relativt kortvariga perioder med ekonomiska problem
3 ☐ Ja, svåra och/eller långvariga perioder med ekonomiska problem

---

Fråga 122 Vilket <u>huvudsakligt</u> yrke eller sysselsättning hade dina föräldrar *under din uppväxttid*?

Försök att lämna en yrkesbeskrivning som noggrannt beskriver arbetsuppgifterna.
I stället för chaufför, skriv t.ex. busschaufför, lastbilschaufför etc.

Exempel: I stället för lärare, skriv t.ex.

F	Ö	R	S	K	O	L	E	L	Ä	R	A	R	E								

Faderns yrke:

Moderns yrke:

Livskvalitet

Fråga 123 **Markera genom att kryssa i en ruta i varje nedanstående grupp, vilket påstående som bäst beskriver Ditt hälsotillstånd i dag.**

a) Rörlighet

1 ☐ Jag går utan svårigheter
2 ☐ Jag kan gå med viss svårighet
3 ☐ Jag är sängliggande

b) Hygien

1 ☐ Jag behöver ingen hjälp med min dagliga hygien, mat eller påklädning
2 ☐ Jag har vissa problem att tvätta eller klä mig själv
3 ☐ Jag kan inte tvätta eller klä mig själv

c) Huvudsakliga aktiviteter: t.ex. arbete, studier, hushållssysslor, familje- och fritidsaktiviteter

1 ☐ Jag klarar mina huvudsakliga aktiviteter
2 ☐ Jag har vissa problem med att klara av mina huvudsakliga aktiviteter
3 ☐ Jag klarar inte av mina huvudsakliga aktiviteter

d) Smärtor/besvär

1 ☐ Jag har varken smärtor eller besvär
2 ☐ Jag har måttliga smärtor eller besvär
3 ☐ Jag har svåra smärtor eller besvär

e) Oro/nedstämdhet

1 ☐ Jag är inte orolig eller nedstämd
2 ☐ Jag är orolig eller nedstämd i viss utsträckning
3 ☐ Jag är i högsta grad orolig eller nedstämd

Fråga 124

Till hjälp för att avgöra hur bra eller dåligt ett hälsotillstånd är, finns den termometer-liknande skalan till höger. På denna har Ditt bästa tänkbara hälsotillstånd markerats med 100 och ditt sämsta tänkbara hälsotillstånd med 0.

Vi vill att Du på denna skala markerar hur bra eller dåligt Ditt hälsotillstånd är, som Du själv bedömer det. Gör detta genom att dra en linje från nedanstående ruta till den punkt på skalan som markerar hur bra eller dåligt ditt nuvarande hälsotillstånd är.

Ditt nuvarande hälsotillstånd

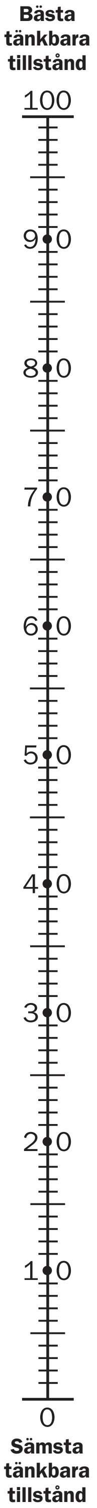

Fråga 125

Tänk dig att Du får reda på att Du har 10 år kvar att leva. I samband med detta får Du välja mellan att leva dessa 10 år i Ditt nuvarande hälsotillstånd, eller att avstå något/några år för att istället leva kortare tid med full hälsa.

Markera med ett kryss (X) på linjen det antal år med full hälsa som Du tycker är likvärdigt med att leva 10 år i Ditt nuvarande hälsotillstånd.

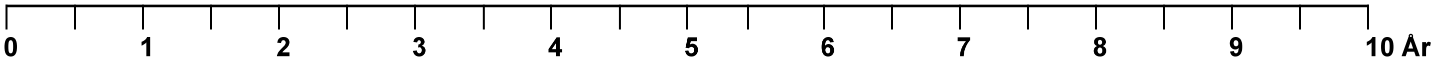

Antal år med full hälsa
(Anser Du att Du för närvarande har full hälsa ska Du kryssa i 10 år)

Vårdutnyttjande

Fråga 126 Har Du under <u>de senaste tre månaderna</u> för egna besvär eller sjukdom ...

	Nej 1	Ja 2
a) varit inlagd på sjukhus?	☐	☐
b) besökt läkare på sjukhusmottning, vårdcentral eller liknande?	☐	☐
c) besökt akutmottagning?	☐	☐
d) besökt distriktssköterska?	☐	☐

Fråga 127 Har Du under <u>de senaste tre månaderna</u> ansett Dig vara i behov av läkarvård, men inte sökt vård?

1 ☐ Nej ———> **Gå till fråga 129**

2 ☐ Ja

Fråga 128 Vilken var orsaken/orsakerna till att Du inte sökte vård?
Du kan ange flera alternativ.

1 ☐ Besvären gick över

1 ☐ Hade inte råd

1 ☐ För långa väntetider

1 ☐ Tycker inte jag kan få någon hjälp

1 ☐ Kom inte fram på telefon

1 ☐ Fick inte tag på någon läkare

1 ☐ Kände inte till någon bra läkare

1 ☐ Hade inte tid

1 ☐ Ville vänta ett tag

1 ☐ Annan orsak. Vad?

____

____

Fråga 129 Vi vill <u>eventuellt</u> återkomma till Dig för en intervju med kompletterande frågor. Om Du går med på det, vänligen fyll i uppgifter om namn, adress och telefonnummer. Om Du inte vill bli kontaktad kryssar Du för Nej och utelämnar namn, adress o.s.v.

1 ☐ Ja, jag kan tänka mig att bli kontaktad för att svara på frågor.

2 ☐ Nej, jag vill inte bli kontaktad

Namn: ______________________________

Adress: ______________________________

Postnummer: ______________ *Postadress:* ______________________________

Telefonnummer: ________ / ______________________________

Fråga 130 **Finns det något övrigt som Du vill påpeka och som handlar om Din hälsa, hälsorisker i Din hemkommun eller saker som Du skulle vilja göra för att Din hemkommun ska bli en bättre kommun att leva och bo i?**

Tack för att du tog dig tid att fylla i frågeblanketten!

SCB-Tryck, Örebro 2004.08 MILJÖMÄRKT Trycksak 341242
