# Supplementary material for: Adult asthma and traffic exposure at residential address, workplace address, and self-reported daily time outdoor in traffic: A two-stage case-control study
Source: BMC Public Health. 2010 Nov 22;10:716. doi: 10.1186/1471-2458-10-716 (PMC3003254; doi:10.1186/1471-2458-10-716)
Supplement: Additional file 2 — Survey1_2004_English translation. English translation of the first survey questionnaire (2004). [file 1471-2458-10-716-S2.PDF]

Public Health in Skåne 2004

Take part and influence the design of future healthcare in Skåne!

How can we improve our preventive work?

How can health be improved in Skåne?

You are one of the people in Skåne who have been invited to take part in this survey. The aim is to increase our knowledge about the health and environment of Skåne residents. The questionnaire you have received includes questions about your health status and factors that are of importance with regard to health.

Why is it important that you answer the questionnaire?
We are conducting this survey in order to obtain a current picture of the health and healthcare contacts of Skåne residents, and of the living and environmental conditions that will be important to future public health in Skåne. Your participation is voluntary. For the results to be accurate, it is important that as many people as possible respond to the survey. When you answer the questionnaire, you make everyone else's participation more meaningful. Your answers will help us in our efforts to formulate various measures to promote health and prevent illness, and to improve health and medical care in Skåne. That's why your responses are very important.

What will happen with your responses?
Naturally your identity and any information about you will be carefully protected and kept secret. All the information that we compile will be presented in table form, making it completely impossible to identify any one individual.

Any questions?
The Department of Social Medicine is responsible for the survey, but Statistics Sweden has been tasked with disseminating and collecting the questionnaires and compiling the results. If there is anything you would like to discuss with us in connection with filling out the form or about the survey otherwise, please feel free to ring us at one of the telephone numbers listed on the next page.

Sincerely,

Kent Karlsson
PhD, Strategist Health Planner,
Health and Medical Care Management
Region Skåne

Farhad Ali Khan
Project Manager
Dept. of Social Medicine
University Hospital MAS

Per-Olof Östergren
Professor, Director
Dept. of Social Medicine
University Hospital MAS

Thanks in advance for your cooperation!

MORE ABOUT THE SURVEY

In order to reduce the number of questions in the survey, population register data such as age, country of birth and citizenship will be obtained from Statistics Sweden's Total Population Register. Information about education levels, occupations and incomes will be obtained from other Statistics Sweden registers. Statistics Sweden will also assign a code to enable various geographical breakdowns. Information concerning doctors' visits, hospital stays and sick leaves will be obtained from the County Council's databases and the Swedish National Social Insurance Board.

Your answers will be protected under the Swedish Confidentiality Act (SFS 1980:100, Chapter 9 § 4) and the Swedish Personal Data Protection Act. This means that everyone who works on the survey will be subject to a confidentiality obligation, and that the gathered data will be presented in tables from which no one individual's answers can be determined. The number at the top of the form will be used to enable Statistics Sweden to determine who has responded and who should receive a reminder.

All identifying information will be removed before Statistics Sweden delivers the material to the Department of Social Medicine. To enable us to follow up the health situation in the future, Statistics Sweden will keep a "key" linking the names and the questionnaire serial numbers for the next 10 years. Only affected Statistics Sweden personnel will have access to these data.

If you have any questions about the survey (background, purpose, how to answer the questions etc.), please feel free to ring: Viveca Flodén, Department of Social Medicine, University Hospital MAS, tel. +46 040-33 28 74 between 1 pm and 2:30 pm, or send an e-mail to fhs2004@skane.se.

If you have any questions about the actual data-gathering process, please feel free to ring: Statistics Sweden, tel. +46 019-17 60 65, or send an e-mail to_fhs2004@scb.se.

How to fill out the questionnaire
Answering the questions will take about 20-30 minutes. The answers will be registered by machine, making it important to fill out the questionnaire in the right way.

• Use a good ballpoint pen with black or dark blue ink. Avoid pencils.

• Tick the boxes that you feel best apply to you. Keep the tick marks inside the boxes, if possible.

Like this ☒

NOT this: ☒

• If you tick the wrong box, fill it in entirely with ink.

Like this: ■
The machine will not be able to read it. Now tick the correct box.

Response time
We would greatly appreciate it if we could receive your completed questionnaire within one week.

Response envelope and postage
Send the questionnaire back in the enclosed envelope. The postage is pre-paid.

Thanks for your cooperation!

Health

Question 1 **How would you rate your current health status in general?**

1 Very good
2 Good
3 Fair
4 Poor
5 Very poor

Question 2 **How tall are you?** Whole cm

cm

Question 3 **How much do you weigh?** Whole kg

kg

Question 4 In the last 14 days, have you been troubled by any of the symptoms or complaints listed below? Tick one box in each row!

	Heavily 1	To some extent 2	To a small extent 3	Not at all 4
a) Pain in shoulders, neck or upper arms				
b) Back pain, backache, hip pain or sciatica				
c) Aches or pains in hands, elbows, legs or knees				
d) Headache or migraine				
e) Anxiety, worry, fears				
f) Tiredness				
g) Insomnia				
h) Eczema or skin rash				
i) Ringing in the ears (tinnitus)				
j) Incontinence (urine leakage)				
k) Recurrent stomach/intestinal problems				

Question 5 Do you have any of the following health problems?

Tick one box in each row!	No 1	Yes, but no symptoms 2	Yes, mild symptoms 3	Yes, severe symptoms 4
a) Allergies				
b) Asthma				
c) Diabetes				
d) High blood pressure				

Question 6 a) In the last <u>14 days</u>, have you been prevented from doing your daily tasks (e.g. working inside or outside the home, leisure activities or the like) because of any illness, injury or other reason?

I Yes

2 No ———> Go to question 7

b) If YES: Are these problems of a more chronic nature? Chronic means that they have been present or are expected to last for 6 or more months.

I Yes

2 No

If you do not work Go to question 9.

Question 7 a) Have you been forced to stay home from work because of illness, injury or other problems <u>in the last year?</u>

I No, not at all in the last year > Go to question 8

2 Yes

b) If YES: How many workdays?
Count only workdays!

workdays

Question 8a) Have you been forced to stay home from work because of illness, injury or other problems in the <u>last two weeks</u>?

I No, not at all during the last two weeks

2 Yes

b) If YES: How many workdays?
Count workdays only!

workdays

Question 9 Do you have any long-term illness, problems following an accident, or any handicap or other debility?

I Yes

2 No

Question 10 Have you <u>ever</u> been forced to stop working, change jobs or change job duties because of illness, injury or symptoms?

I Yes, changed jobs/job duties

2 Yes, stopped working

3 Yes, first changed jobs/job duties and then stopped working

4 No

Question 11 The questions here have to do with how you feel, and how you have been doing <u>over the last four weeks</u>. For each question, choose the answer that best describes how you have been feeling.

How much of the time over <u>the last four weeks…</u>	All the time	Most of the time	A good bit of the time	Some of the time	A little of the time	None of the time
	1	2	3	4	5	6
a) did you feel full of pep?						
b) have you been a very nervous person?						
c) have you felt so down in the dumps that nothing could cheer you up?						
d) have you felt calm and peaceful?						
e) did you have a lot of energy?						
f) have you felt downhearted and blue?						
g) did you feel worn out?						
h) have you been a happy person?						
i) did you feel tired?						

Medications

Question 12 Over the last three months, have you used any of the following drugs or preparations?

Tick one box in each row!	No<br>1	Yes, in the last three months<br>2	Yes, in the last 14 days<br>3
a) Blood pressure medication			
b) Medicine for asthma/allergy			
c) Non-prescription pain medicine			
d) Prescription pain medicine			
e) Antibiotics			
f) Sleeping aid			
g) Calmative medication			
h) Anti-depressant			
i) Ulcer medication			
j) Oestrogen preparation			
k) Vitamins/Minerals			
l) Natural medicine			
m) Other ______			

Question 13 Medication use

Tick one box in each row!	Yes<br>1	No<br>2	Don’t take medicine<br>3
a) Do you sometimes forget to take your medication?			
b) Are you always careful to take your medication?			
c) If you feel better, do you sometimes decide not to take your medication?			
d) If you feel worse when you take your medication, do you sometimes decide not to take it?			

Question 14 a) In the last three months, have you ever been given a prescription but not filled it at the pharmacy?

1 Yes, one time

2 Yes, several times

3 No ———> Go to Question 15

b) If YES: Why did you not pick up your medicine?

You can tick more than one alternative.

1 I got better

1 I had enough medicine

1 I couldn’t afford it

1 It was too far to the pharmacy

1 The medicine still doesn’t help

1 Other

Sleep and well-being

Question 15 Do you think you get enough sleep to feel rested?

1 Yes, as a rule
2 Yes, but not often enough
3 No, never or almost never

Question 16 Over the last 14 days, have you been troubled by insomnia and, if so, how troubled were you?

1 Yes, very troubled
2 Yes, a little troubled
3 No

Question 17 Do you feel stressed in your daily life?

1 Yes, often
2 Yes, sometimes
3 No, (almost never)

Question 18 Over the last month, have you often felt troubled because you were feeling down, depressed or resigned?

1 Yes
2 No

Question 19 Over the last month, have you often felt troubled because you have felt uninterested or have derived less enjoyment than usual from doing various activities?

1 Yes

2 No

Question 20 Over the last few weeks, have you been able to concentrate on whatever you´re doing?

1 Better than usual
2 Same as usual
3 Worse than usual
4 Much worse than usual

Question 21 Have you lost much sleep over worry the last few weeks?

1 Not at all
2 No more than usual
3 Rather more than usual
4 Much more than usual

Question 22 Have you felt that you are playing a useful part in things over the last few weeks?

1 More than usual
2 Same as usual
3 Less than usual
4 Much less than usual

Question 23 Have you felt capable of making decisions about things over the last few weeks?

1 Better than usual
2 Same as usual
3 Worse than usual
4 Much worse than usual

Question 24 Have you felt constantly under strain over the last few weeks?

1 Not at all
2 No more than usual
3 Rather more than usual
4 Much more than usual

Question 25 Over the last few weeks, have you felt that you couldn't overcome your difficulties?

1 Not at all
2 No more than usual
3 Rather more than usual
4 Much more than usual

Question 26 Over the last few weeks, have you been able to enjoy your normal day-to-day activities?

1 More than usual
2 Same as usual
3 Less than usual
4 Much less than usual

Question 27 Have you been able to face up to your problems over the last few weeks?

1 Better than usual
2 Same as usual

3 Worse than usual
4 Much worse than usual

Question 28 Have you been feeling unhappy and depressed over the last few weeks?

1 Not at all
2 No more than usual
3 Rather more than usual
4 Much more than usual

Question 29 Have you been loosing confidence in yourself over the last few weeks?

I Not at all
2 No more than usual
3 Rather more than usual
4 Much more than usual

Question 30 Have you been thinking of yourself as a worthless person over the last few weeks?

I Not at all
2 No more than usual
3 Rather more than usual
4 Much more than usual

Question 31 Have you been feeling reasonably happy, all things considered, over the last few weeks?

I More than usual
2 Same as usual
3 Less than usual
4 Much less than usual

Question 32 How do you feel right now, <u>physically and mentally</u>, considering your health and well-being?

Tick the appropriate box between 1 and 7
(1 = Very bad, 7 = Very good)

1 2 3 4 5 6 7

Dental health

Question 33 When was the last time you visited a dentist/dental hygienist?

I Less than a year ago
2 I-2 years ago
3 3-5 years ago
4 More than five years ago
I have never been to a dentist/dental hygienist

Question 34 How would you rate your dental health?

I Very good
2 Fairly good
3 Neither good nor bad
4 Fairly bad
Very bad

Question 35 <u>Over the last three months,</u> have you considered yourself to be in need of dental care but not sought care?

I No ———> Go to question 37
2 Yes

Question 36 What were the reasons why you did not seek care?
You can tick more than one alternative.

I The problem went away
I Couldn't afford it
I Was afraid to go (fear of dentists)
I Didn't have the time
I Another reason. What?

Question 37 Have you ever had any of the following complaints?

Tick one box in each row!	No<br>1	Yes, mild<br>2	Yes, severe<br>3
a) Problems with cavities (holes in teeth)			
b) Bleeding gums			
c) Loose teeth			
d) Difficulty chewing			
e) Sensitive teeth			
f) Gnashing/grinding of teeth			

Living habits

Question 38 How much have you moved about and exerted yourself physically in your spare time over the last 12 months?

If your activity level varies between, for example, summer and winter, try to estimate an average. Tick only one alternative!

1 Regular exercise and workouts.
You engage in, for instance, jogging, swimming, tennis, badminton, calisthenics or the like at least 3 times a week. Each session lasts at least 30 minutes.

2 Moderate regular exercise during spare time
You exercise regularly 1-2 times per week for at least 30 minutes each time, jogging, swimming, tennis, badminton or some other activity that causes you to perspire.

3 Moderate exercise in your spare time
You walk, cycle or move about in some way for at least 2 hours a week, usually without perspiring. Includes walking or cycling to and from work, other walks, ordinary gardening work, fishing, table tennis, bowling.

4 Sedentary spare time
You spend most of your spare time reading, watching TV or movies, or doing other sedentary activities. You walk, cycle or move about in some other way less than 2 hours per week.

Question 39 In a normal week, how much time do you spend engaged in strenuous physical activities that get you heated up, such as fast walking, gardening, heavier household chores, cycling, swimming?Q

This may vary during the year, but try to estimate some sort of average. Tick one alternative!

1 5 hours per week or more
2 More than 3 but less than 5 hours per week
3 Between 1 and 3 hours per week
4 No more than 1 hour per week
5 Not at all
6 Don't know/can't decide

Question 40 Do you want to increase your physical activity?

1 Yes, and I think I can do it by myself
2 Yes, but I need support
3 No

Question 41 How often do you eat green and root vegetables?

This includes all types of green vegetables, legumes and root vegetables except potatoes (fresh, frozen, preserved, stewed, juices, soups etc.)

This may vary during the year, but try to estimate some sort of average. Tick one alternative!

1 3 times per day or more
2 2 times per day
3 1 time per day
4 5-6 times per week
5 3-4 times per week
6 1-2 times per week
7 A few times a month or never

Question 42 How often do you eat fruit or berries?

This includes all types of fruits and berries (fresh, frozen, preserved, juices, compotes etc.)

This may vary during the year, but try to estimate some sort of average. Tick one alternative!

1 3 times per day or more
2 2 times per day
3 1 time per day
4 5-6 times per week
5 3-4 times per week
6 1-2 times per week
7 A few times per month or never

Question 43 What type of spread do you usually use on sandwiches?

Tick one alternative, the most common one!

1 Butter/table margarine, 80% fat such as Bregott, Flora, Linnéa
2 Household margarine (margarine in foil) such as Milda, HushållsEve, Ädel
3 Medium margarine, 60% fat such as Bregott Mellan, Runda Bords
4 Light margarine, 30-40% fat such as Lätta, Lätt&Lagom, Becel, LättLätt, Gaio
5 I don't use spreads on sandwiches/don't eat sandwiches

Question 44 In choosing what you eat, how important is it that the food be "nutritious" or "healthy"?

1 It's very important
2 It's fairly important
3 It's not important
4 Don't know/no opinion

Question 45 Have you changed or tried to change your eating habits in order to eat healthier over the last year?

1 Yes, I have started eating healthier
2 Yes, I have tried but failed
3 No, but I've thought about it
4 No, never
5 No, I think that I already eat healthy
6 I had already changed my eating habits earlier

Question 46 a) Do you smoke?

1 Yes, every day
2 Yes, but not every day
3 No — Go to question 47

b) If YES, EVERY DAY: How much do you smoke on average?

cigarettes a day

cigarillos a day

cigars a day

grams of pipe tobacco per week

Go to question 48

Question 47 a) Have you previously smoked every day for <u>at least 6 months</u>?

1 No ———> Go to question 49
2 Yes

b) If YES: How long ago did you stop smoking every day?

1 Less than 6 months ago
2 Between 6 and 12 months ago
3 Over 1 year ago

indicate year

c) How did you quit smoking?

1 By myself, without nicotine replacement
2 By myself, with the help of nicotine replacement
3 I got professional help
4 Began using chewing tobacco instead

Question 48 How old were you when you started smoking every day?

years old

Question 49 Do you want to quit smoking?

1 I don't smoke
2 Yes, and I think I can do it by myself
3 Yes, but I need support
4 No

Question 50 How often do you spend time in places where other people are smoking or have recently smoked?

Tick one box in each row!	Everyday	One or more times times a week	One or more times a month	Less often or never
	1	2	3	4
a) At home				
b) At work				
c) At a café, bar or restaurant				
d) Somewhere else indoors such as at a friend's, in the car etc.				

Question 51 Do you chew tobacco every day?

I No

2 Yes —> Go to question 54

Question 52 Do you chew tobacco on occasion?

I No

2 Yes

Question 53 a) Have you previously chewed tobacco every day for <u>at least 6 months</u>?

I No ———> Go to question 55

2 Yes

b) If YES: How long ago did you stop chewing tobacco every day?

I Less than 6 months ago

2 Between 6 and 12 months ago

3 Over 1 year ago,

indicate year

Question 54 How old were you when you started chewing tobacco every day?

years old

Question 55 Do you want to stop chewing tobacco?

I I don't chew tobacco

2 Yes, and I think I can do it by myself

3 Yes, but I need support

4 No

Here are some questions about your alcohol use. We would be grateful if you could answer them as accurately and honestly as possible by ticking the alternative that applies to you. "Alcohol" refers to light beer, medium/strong beer, hard cider, wine, fortified wine, and hard liquor.

A "glass" means

50 cl light beer	33 cl strong beer	1-15 cl white or red wine	5-8 cl fortified wine	4 cl hard liquor (such as whiskey)

Question 56 How often have you drunk alcohol over the last 12 months?

I 4 times/week or more

2 2-3 times/week

3 2-4 times/month

4 1 time/month or less often

5 Never ———> Go to question 61

Question 57 How many "glasses" (see example) do you drink on a typical day in which you drink alcohol?

I 1-2

2 3-4

3 5-6

4 7-9

5 10 or more

Question 58 How often do you drink six or more "glasses" at one time?

I Daily or almost everyday

2 Every week

3 Every month

4 Less often than once per month

5 Never

Question 59 How often over the last 12 months have you drunk so much alcohol that you became intoxicated?

I Daily or almost everyday

2 Several times/week

3 1 time/week

4 2-3 times/month

5 1 time/month

One or a few times every six months

Less often or never

Question 60 Do you want to reduce your alcohol consumption?

1 Yes, and I think I can do it by myself

2 Yes, but I need support

3 No

Question 61 In the last 12 months, have you brought alcohol home with you to Sweden after travelling abroad?

1 I have not been abroad in the last 12 months

2 Yes

3 No

Question 62 In the last 12 months, have you purchased brand name hard liquor from a private person at a lower price than you would have paid at a Swedish Alcohol Retail Monopoly (Systembolaget) shop?

"Brand name hard liquor" refers to bottles bearing a label from an official distiller.

1 Yes, regularly

2 Yes, several times

3 Yes, a few times

4 No, never

Question 63 Have you ever smoked hashish or marijuana?

1 No

2 Yes, in the last month

3 Yes, in the last year

4 Yes, more than a year ago

Question 64 In the last 12 months, have you bought a lottery ticket or bet money gambling?

"Gambling" refers to playing Trisslott (scratch cards), Bingolotto, casino gambling, football pools, betting on horses or the like

1 No ———> Go to Question 67

2 Yes

Question 65 How much money have you bet on gambling in the last 7 days?

I have bet

crowns.

I have not bet any money in the last 7 days

Question 66 How many times over the last 12 months have you...

Tick one box in each row!	Never<br>1	1-2 times<br>2	3 times or more<br>3
a) tried to reduce your gambling?			
b) felt restless and irritable if you couldn't gamble?			
c) lied about how much you've been gambling?			

Social relationships

Question 67 Do you feel that you have one or more people who can give you proper personal support to cope with life's stress and problems?

1 Yes, absolutely

2 Yes, probably

3 Not entirely sure

4 No

Question 68 Can you get help from one or more people in the event of illness or practical problems (borrowing small items, help with repairs, help in writing a letter, getting advice or information)?

1 Yes, without a doubt

2 Yes, probably

3 No, probably not

4 No, not at all

Question 69 Over the last 12 months, have you...

You can tick multiple boxes!

1 participated in a study circle/course where you work
1 participated in a study circle/course in your spare time
1 participated in a trade union meeting
1 participated in a meeting of some other association
1 been to the theatre/cinema
1 been to an art exhibit
1 participated in a religious gathering
1 been to a sporting event
1 written a letter to the editor of a newspaper/magazine
1 participated in a demonstration of any kind
1 attended a public gathering such as a nightclub, dance party or the like
1 participated in a major family gathering
1 attended a private party at someone's home
1 none of the above

Question 70 Do you feel rooted and that you have a strong sense of attachment to the place where you live?

1 To a large extent
2 To a certain extent
3 Not particularly
4 Not at all

Question 71 Do you feel rooted and that you have a strong sense of attachment to your workmates?

1 I don't work
2 I have no workmates
3 To a large extent
4 To some extent
5 Not particularly
6 Not at all

Question 72 Indicate your attitude toward the following statements:

a) Most people would exploit you if they got the chance.

1 Don't agree at all
2 Don't agree
3 Agree
4 Agree completely

b) Most people are basically fair.

1 Don't agree at all
2 Don't agree
3 Agree
4 Agree completely

c) One can trust most people.

1 Don't agree at all
2 Don't agree
3 Agree
4 Agree completely

d) One cannot be too careful when dealing with other people.

1 Don't agree at all
2 Don't agree
3 Agree
4 Agree completely

Question 73 In the last 12 months, have you been personally attacked because of your race or skin colour?

1 Yes, through something someone said or wrote to you
1 Yes, someone physically attacked your person or destroyed or vandalized something belonging to you
1 Yes, in some other way
1 No

Question 74 a) Do you think that there are employers in Sweden who would refuse to give someone a job because of his or her race, skin colour, religious affiliation or cultural background?

1 None or very few
2 Less than half
3 About half
4 Most

b) Which of the following do you consider among the most important goals for Swedish politics? (Choose a maximum of two alternatives):

1 Maintain law and order in the country
1 Give people more influence on political decisions
1 Fight rising prices
1 Defend freedom of expression
1 Don't know/no opinion

Threats of violence

Question 75 In the last 12 months, have you been subject to a threat or threats of violence that were so dangerous or serious that you became frightened?

1 Yes

2 No

Question 76 a) Have you been subjected to physical violence at any time in the last 12 months?

1 Yes

2 No ———>Go to Question 77

b) If YES: Where did it happen?
You can tick more than one alternative!

1 In the workplace/at work/school

1 At home

1 At someone else's house/neighbourhood

1 In a public place/place of amusement/on the train, bus, underground

1 Somewhere else

c) If YES: Did this violence cause enough bodily harm that you had to seek medical care?

1 Yes

2 No

Confidence in social institutions

Question 77 What confidence do you have in the following institutions in society?

Tick one box in each row!	Very high	Fairly high	Not particularly high	None at all	No opinion
	1	2	3	4	5
a) Healthcare system					
b) Schools					
c) The police					
d) Social Services					
e) Swedish Public Employment Agency					
f) Social Insurance system					
g) Courts					
h) Swedish Parliament					
i) Politicians in your county council					
j) Politicians in your municipality					
k) Mass media, TV, Newspapers, magazines					

Work, employment and the economy

Question 78 a) What is/was your occupation or your job duties?

If you are not gainfully employed, indicate the primary profession/job duties that you have had.
If you have never been gainfully employed go to Question 79.

Try to provide an occupation that describes your main job duties specifically. For instance, instead of “driver”, write “bus driver”, “lorry driver” etc.

Example: Instead of “teacher” write, for instance:

P	R	E	S	C	H	O	O	L		T	E	A	C	H	E	R					

Occupation:

Describe your primary job duties. For instance, if you are a project manager, specify that you are responsible for improving the work environment in geriatric care, responsible for developing the system used to shorten waiting times at call centres etc.

b) Describe your primary job duties as specifically as possible.

Question 79 What is your current employment status?

You can tick more than one alternative!

1 Employed % of full time

1 On leave of absence/parental leave

1 Student, apprentice

1 State unemployment programme

1 Unemployed

1 Old age pensioner

1 Retired under collective agreement

1 Early retirement, retired due to illness

1 On long-term sick leave (more than 3 months)

1 Keeping private household

1 Other. What? ___

Here are some questions for you if you are gainfully employed (even if you are on sick leave or parental leave / leave of absence). Others please proceed to Question 96.

Question 80 Who is your primary employer? Tick one alternative!

1 Privately-owned company

2 Government agency/state-owned corporation or department

3 Region/County Council/ County council-owned corporation

4 Municipality/Municipality-owned corporation

5 Have my own business

6 Other. What

Question 81 a) Have you been gainfully employed in the last 12 months?

1 No

2 Yes

b) If YES: How many months in the last 12 months have you been gainfully employed?

months

Question 82 What is your current form of employment?
Tick one alternative!

1 Private business owner
2 Permanent position (conditional tenure)
3 Temporary position
4 Project-based position
5 Hourly employee
6 Employed by temporary employment agency or staffing company
7 Employed on trial basis
8 Other position

Question 83 If you are employed or a private business owner, what is your employment status?

1 Employed
2 Private business owner with no employees
3 Private business owner with 1-9 employees
4 Private business owner with 10 or more employees

Question 84 a) Have you been involuntarily unemployed at any time over the last three years?

1 Yes
2 No

b) If YES: Approximately how long in total have you been unemployed over the last three years?

months

Question 85 Are you worried about losing your job over the next year?

1 Not at all worried
2 Not particularly worried
3 Fairly worried
4 Very worried

Question 86 Is the company/workplace where you currently work the place where you want to work in the future?

1 Yes
2 No

Question 87 Is your current occupation the occupation you want to have in the future?

1 Yes
2 No

Question 88 Can you adjust your job if you are feeling listless, are in pain, have a cold etc.?

1 Seldom or never
2 Sometimes
3 Often

Question 89 Because of your job, can it be difficult for you to stay home if you are sick for one or two days?

1 Seldom or never
2 Sometimes
3 Often

Question 90 How many times in the last 12 months have you gone to work even though you really should have been on sick leave because of your health?

1 Not once
2 One time
3 Several times
4 Many times

Work environment

If you have not been gainfully employed over the last 12 months, go to Question 96.

Question 91 Do you agree with the following statements?

a) My job requires that I learn new things.

1 Strongly Disagree
2 Disagree
3 Agree
4 Strongly Agree

b) My job involves a lot of repetitive work.

1 Strongly Disagree
2 Disagree
3 Agree
4 Strongly Agree

c) My job requires me to be creative.

1 Strongly Disagree
2 Disagree
3 Agree
4 Strongly Agree

d) My job allows me to make a lot of decisions on my own.

1 Strongly Disagree

2 Disagree

3 Agree

4 Strongly Agree

e) My job requires a high level of skill.

1 Strongly Disagree

2 Disagree

3 Agree

4 Strongly Agree

f) On my job, I have very little freedom to decide how I do my work.

1 Strongly Disagree

2 Disagree

3 Agree

4 Strongly Agree

g) I get to do a variety of different things on my job.

1 Strongly Disagree

2 Disagree

3 Agree

4 Strongly Agree

h) I have a lot of say about what happens on my job.

1 Strongly Disagree

2 Disagree

3 Agree

4 Strongly Agree

i) I have an oppertunity to develop my own special abilities.

1 Strongly Disagree

2 Disagree

3 Agree

4 Strongly Agree

j) My job requires working very fast.

1 Strongly Disagree

2 Disagree

3 Agree

4 Strongly Agree

k) My job requires working very hard.

1 Strongly Disagree

2 Disagree

3 Agree

4 Strongly Agree

l) I am not asked to do an excessive amount of work.

1 Strongly Disagree

2 Disagree

3 Agree

4 Strongly Agree

m) I have enough time to get the job done.

1 Strongly Disagree

2 Disagree

3 Agree

4 Strongly Agree

n) I am free from conflicting demands that others make.

1 Strongly Disagree

2 Disagree

3 Agree

4 Strongly Agree

o) My job requires long periods of intense concentration on the task.

1 Strongly Disagree

2 Disagree

3 Agree

4 Strongly Agree

p) My tasks are often interrupted before they can be completed, requireing attention at at later time.

1 Strongly Disagree

2 Disagree

3 Agree

4 Strongly Agree

q) My job is very hectic.

1 Strongly Disagree

2 Disagree

3 Agree

4 Strongly Agree

r) Waiting on work from other people or departments often slows me down on my job.

1 Strongly Disagree
2 Disagree
3 Agree
4 Strongly Agree

s) My supervisor is concerned about the welfare of those under him.

1 I have/had no supervisor
2 Strongly Disagree
3 Disagree
4 Agree
5 Strongly Agree

t) My supervisor pays attention to what I am saying.

1 I have/had no supervisor
2 Strongly Disagree
3 Disagree
4 Agree
5 Strongly Agree

u) My supervisor is helpful in getting the job done.

1 I have/had no supervisor
2 Strongly Disagree
3 Disagree
4 Agree
5 Strongly Agree

v) My supervisor is successful in getting people to work together.

1 I have/had no supervisor
2 Strongly Disagree
3 Disagree
4 Agree
5 Strongly Agree

w) People I work with are competent in doing their jobs.

1 I have/had no coworkers
2 Strongly Disagree
3 Disagree
4 Agree
5 Strongly Agree

x) People I work with take a personal interest in me.

1 I have/had no coworkers
2 Strongly Disagree
3 Disagree
4 Agree
5 Strongly Agree

y) People I work with are friendly.

1 I have/had no coworkers
2 Strongly Disagree
3 Disagree
4 Agree
5 Strongly Agree

z) People I work with are helpful in getting the job done.

1 I have/had no coworkers
2 Strongly Disagree
3 Disagree
4 Agree
5 Strongly Agree

Question 92 a) Do you ever work overtime at your job?

1 Never work overtime
2 Work overtime several times a month
3 Often work overtime
4 Do not have regulated work hours

b) If you WORK OVERTIME: Indicate your average number of overtime hours per week

hours per week

c) If you WORK OVERTIME: Can you usually decide for yourself when you want to work your overtime?

1 Yes
2 No

d) If you WORK OVERTIME: Do you get any sort of overtime compensation?

1 Yes
2 No

Question 93 How often do you encounter the following things at work?
Tick one box in each row!

	Everyday 1	Several days 2	Less often 3	Never a week 4
a) Noise (you have to raise your voice in conversation)				
b) Chemical preparations, vapours, gases				
c) Repetitive and monotonous work motions				
d) Heavy lifting (More than 20 kg/lift)				
e) Harassment or persecution				

Q 94 a) Does your employer offer any type of compensation for exercise or workouts?
You can tick more than one alternative!

1 No, there are no such opportunities
1 N/A
1 Opportunity to workout/exercise during work hours, e.g. fitness hour
1 Subsidized gym card, free swimming or the like
1 Other opportunities to work out, such as?

Q 94 b) Do you take advantage of this/these benefits?

1 Yes, often
2 Yes, sometimes
3 No, never

Q 95 a) How do you usually get to work?
You can tick more than one alternative.

1 Walk
1 Cycle
1 Car
1 Bus
1 Train
1 Other

Q 95 b) How long does it take to get to work (one way)?

1 Less than 15 minutes
2 15-30 minutes
3 30-60 minutes
4 1-1.5 hours
5 1.5-2 hours
6 More than2 hours

Question 96 To what extent do you think that the following factors affect sick leaves in Sweden?

	Very large 1	Fairly large 2	Not especially large 3	None 4	No opinion 5
a) Stressful working conditions					
b) Stresses outside of work					
c) Compensation levels while on sick leave					
d) Doctors' sick leave practices					
e) The way the Social Insurance system works					

Question 97 What importance do you think the following factors could have in terms of your own risk of being put on sick leave?

	Very high	Fairly high	Not especially high	None	No opinion/N/A
	1	2	3	4	5
a) A bad work environment due to hazardous substances, noise, stressful working positions/lifting					
b) Excessive psychological demands on the job					
c) Too little influence over work situation					
d) Poor support from the supervisors					
e) Bad mood among workmates					
f) Worries about losing job					
g) Problems in family/personal life					
h) Hard to get time to treat, address problems					

Home, household and living environment

Question 98 What type of residence do you live in?

1 Own house/row house
2 Cooperative/condominium
3 Flat w/right of tenancy
4 Lodger/student housing/room
5 Other

Question 99 What is the floor area of your residence?
Estimate if you don't know exactly

square metres

Question 100 How many people live in your family dwelling?

person/people

Question 101 How many rooms are there in your residence?
(not counting the kitchen)

rooms

Question 102 How long have you lived in your current residence?
Indicate number of years

years

Question 103 With whom do you share your residence, i.e. live together for the greater part of the week? You can tick more than one alternative.

1 No one
1 Parents/siblings
1 Spouse/cohabitant/partner
1 Other adults
1 Children ———> How old are the children who live at home?

1 0-6
1 7-12
1 13-17
1 18 or older

Question 104 How many hours a week do you spend working at home (non-occupational work)?
E.g. shopping, cooking, bookkeeping cleaning, watching children, maintaining car, house, garden

1 0-2 hours/week
2 3-10 hours/week
3 11-20 hours/week
4 21-30 hours/week
5 31 hours/week or more

Question 105 a) Do you have any elderly, sick or functionally-impaired relatives whom you need to help with daily chores, watch or care for?

1 No———> Go to Question 106
2 Yes

b) If YES: How many hours of restriction/work does this involve for you per week?

hours per week

Question 106 Are there any green areas (large parks or the like) or forested areas within a 5-10 minute walk from where you live?

1 Yes

2 No

3 Don't know

Question 107 How safe and comfortable do you feel when walking alone in your neighbourhood after dark?

1 Very safe

2 Fairly safe

3 Rather unsafe

4 Very unsafe

5 I never go out alone after dark

Question 108 How happy are you with the area where you live?

1 Very happy

2 Fairly happy

3 Fairly unhappy

4 Very unhappy

5 Don't know/ N/A

Question 109 Thinking about the area where you live, do you feel that...

Tick one box in each row!	Yes<br>1	No<br>2	No opinion<br>3
a) the services are good, e.g. proximity to shops, bank, post office, childcare?			
b) the cultural offering is good, e.g. proximity to library, cinema, theatre?			
c) the leisure opportunities are good, e.g. proximity to swimming pool, sport facilities, exercise circuits?			
d) the public transport & communication system is satisfactory?			
e) there is disruptive noise such as traffic/airplane noise, industries, restaurants, neighbours, fan noise?			
f) there is bothersome air pollution such as exhaust gases, odours, dust, soot?			

Question 110 In the last 3 months, have you been troubled by any of the following in or near your residence?

	Yes, at least once a day<br>1	Yes, at least once a week<br>2	Yes, less often<br>3	No, never<br>4
a) Noise from neighbours				
b) Road traffic noise				
c) Train noise				
d) Airplane noise				
e) Car exhaust				
e) Wood smoke				
e) Odours from industries				

Question 111 Does traffic noise (road, train or air traffic) subject your residence to any of the following disturbances?

	Yes, at least once a day<br>1	Yes, at least once a week<br>2	Yes, less often<br>3	No, never<br>4
a) Hard to hear radio/TV				
b) Talking on phone hampered				
c) Normal conversation hampered				
d) Rest/relaxation hampered				
e) Hard to get to sleep				
f) I get awakened				

Background data

Question 112 What year were you born?

19_ _

Question 113 Are you a man or a woman?

1 Man
2 Woman

Question 114 What is your civil status?

1 Married/cohabitant
2 Unmarried
3 Divorced
4 Widow/widower

Question 115 a) Were you born in Sweden?

1 Yes ———> Go to Question 116
2 No

b) If NO: In what country were you born?

c) If NO: When did you move to Sweden? Year:

Question 116 a) Were your parents born in Sweden?

1 Yes, both ———> Go to Question 117
2 No, one of my parents was born in another country
3 Both parents were born in another country

b) IF EITHER WAS BORN ABROAD: In which country or countries?

Father's country of birth:

Mother's country of birth:

Question 117 What is the main language used in your household?

1 Swedish

2 Other

3 Swedish and another language equally

Question 118 What is the highest level of education you have completed?

If you are a student, tick your current level.

Tick just one!

1 Elementary or nine-year compulsory school

2 Lower secondary school or girls school

3 2-year upper secondary or vocational school

4 3-4-year upper secondary school

5 University or college, less than 3 years (less than 100 credits)

6 University or college, 3 years or more (120 credits or more)

7 Other education. What?

Question 119 In the last 12 months, how often have you had difficulty paying your bills (rent, electricity, telephone, interest, loan instalments, insurance etc.)?

1 Every month

2 About half the months of the year

3 In a few isolated instances

4 Never

Question 120 If you were suddenly faced with an unforeseen situation in which you had to come up with 14,000 kronor in one week, could you do so?

1 Yes

2 No

Question 121 Did your family face financial difficulties during your childhood?

1 No, no noteworthy financial problems

2 Yes, mild and/or relatively short periods of financial problems

3 Yes, severe and/or long-term periods of financial problems

Question 122 What were your parents' primary jobs or occupations during your childhood?

Try to provide an occupation that describes their main job duties specifically. For instance, instead of "driver", write "bus driver", "lorry driver" etc.

Example.: Instead of "teacher" write, for instance:

P	R	E	S	C	H	O	O	L		T	E	A	C	H	E	R					

Father's occupation:

Mother's occupation:

Question 123 Indicate the statement that best describes your current health status by ticking a box in each of the groups below.

a) Mobility

1 I can walk without difficulty
2 I can walk with some difficulty
3 I am bedridden

b) Hygiene

1 I need no help with my daily hygiene, food or clothing
2 I have some problems washing or dressing myself
3 I cannot wash or dress myself

c) Main activities such as work, studies, household chores, family and leisure activities

1 I can manage my main activities
2 I have some problems in managing my main activities
3 I cannot manage my main activities

d) Pains/complaints

1 I have neither pains nor complaints
2 I have moderate pains or complaints
3 I have severe pains or complaints

e) Worry/depression

1 I am neither worried nor depressed
2 I am worried and depressed to some extent
3 I am deeply worried or depressed

Best conceivable status

100

Question 124

To help you determine how good or bad your health status is, we have provided a thermometer-like scale to the right. On the scale, your best conceivable health status would rate 100, while your worst conceivable health status would rate 0.

90

80

70

Please mark this scale to indicate how good or bad your health status is in your own assessment. Do this by drawing a line from the box below to the point on the scale that indicates how good or bad your current health status is.

60

Your current

health status

50

40

30

20

10

0

Worst conceivable status

Question 125

Imagine finding out that you have 10 years left to live. You are also given a choice between living those 10 years with your current health status or dying one or more years earlier in order to instead live a shorter time in full health.

Put an X on the line to indicate the number of years with full health you think would be equivalent to living for 10 years with your current health status

0 1 2 3 4 5 6 7 8 9 10 years

Number of years with full health
(If you currently consider yourself to be fully healthy, put your X at 10 years)

Use of Care

Question 126 Due to personal problems or illness, over the last three months have you...

	No 1	Yes 2
a) been admitted to a hospital?		
b) visited a doctor at a hospital clinic, care centre or the like?		
c) visited an emergency room?		
d) visited a district nurse?		

Question 127 Over the last three months, have you considered yourself to be in need of medical care but did not seek it?

1 No ———> Go to Question 129

2 Yes

Question 128 What is/are the reason(s) why you did not seek care?
You can tick more than one alternative.

1 The problem went away

1 Couldn’t afford it

1 Waiting times are too long

1 Didn’t think I could get any help

1 Didn’t get through on the phone

1 Couldn’t get in touch with a doctor

1 Didn’t know of any good doctors

1 Didn’t have the time

1 Wanted to wait awhile

1 Other reason. What?

Question 129 We may want to get back to you for an interview with supplemental questions. If you consent to participate, please enter your name, address and telephone number. If you do not wish to be contacted, tick “No” and leave out your name, address etc.

1 Yes, I would be open to being contacted to answer questions.

2 No, I do not wish to be contacted.

Name: _ Address:

Postal code: Postal address:

Telephone no.: __/______

Question 130 **Is there anything else you would like to point out with regard to your health, health risks in your home municipality, or things that you would do to make your home municipality a better one in which to live and spend time?**

Thank you for taking the time to fill out the question form!
