# Supplementary material for: Adult asthma and traffic exposure at residential address, workplace address, and self-reported daily time outdoor in traffic: A two-stage case-control study
Source: BMC Public Health. 2010 Nov 22;10:716. doi: 10.1186/1471-2458-10-716 (PMC3003254; doi:10.1186/1471-2458-10-716)
Supplement: Additional file 3 — Survey2_2005_Swedish orginal. The Swedish original questionnaire for the second survey (2005). [file 1471-2458-10-716-S3.PDF]

Kodnummer (ifylles av YMK)

Bakgrundsdata

Fråga 1. Vilket år är Du född?

1	9		

Fråga 2. Är Du kvinna eller man?

☐ Kvinna

☐ Man

Fråga 3. Vilket datum fyller Du i enkäten?

2 0 0 _ - _ _ - _ _

år månad dag

Uppväxtförhållanden

Fråga 4. Var har Du tidigare bott i Ditt liv (gäller alltså inte nuvarande bostadsort)?
Du kan ange flera alternativ på varje rad.

Din ålder	Storstad (ex Stockholm, Göteborg, Malmö)	Mindre stad (ex Lund, Kristianstad, Helsingborg, Trelleborg)	Tätort (ex Hässleholm, Ystad, Simrishamn, Höör)	Landsbygd (samhälle med mindre än 200 invånare)
0 - 6 år	☐	☐	☐	☐
7 - 16 år	☐	☐	☐	☐
17 - 30 år	☐	☐	☐	☐
31 - 65 år	☐	☐	☐	☐

Nuvarande bostad

Fråga 5. Hur länge har Du bott i Din nuvarande bostad?

☐☐ år

Fråga 6. Hur många våningar har huset Du bor i?

☐☐ våningar

Fråga 7. Vilket våningsplan bor Du på? Gäller endast om Du bor i flerfamiljshus.
(Bottenvåningen = våning 0, en trappa upp = våning 1, osv.)

Våning ☐☐

Fråga 8. När är huset som Du bor i byggt?

- ☐ Före 1941
- ☐ 1941 - 1960
- ☐ 1961 - 1975
- ☐ 1976 - 1985
- ☐ Efter 1985
- ☐ Vet ej

Fråga 9.

a) Har Din bostad fönster mot (inom ett avstånd på 50 meter):

- ☐ gata?
- ☐ järnväg?
- ☐ industriområde?
- ☐ gård, trädgård, vatten eller grönområde?
- ☐ Något annat, vad? ____________________

b) Vilken trafikintensitet är det på den största gata Du kan se från något fönster i Din bostad (inom ett avstånd på 50 meter)?

- ☐ 0 - 1 fordon/minut
- ☐ 2 - 5 fordon/minut
- ☐ 6 - 10 fordon/minut
- ☐ Fler än 10 fordon/minut
- ☐ Kan inte se någon gata inom 50 meter

Fråga 10.

a) Har Ditt sovrum fönster mot (inom ett avstånd på 50 meter):

- ☐ gata?
- ☐ järnväg?
- ☐ industriområde?
- ☐ gård, trädgård, vatten eller grönområde?
- ☐ Något annat, vad? ____________________

b) Vilken trafikintensitet är det på den <u>största</u> gata Du kan se från Ditt <u>sovrumsfönster</u> (inom ett avstånd på 50 meter)?

- ☐ 0 - 1 fordon/minut
- ☐ 2 - 5 fordon/minut
- ☐ 6 - 10 fordon/minut
- ☐ Fler än 10 fordon/minut
- ☐ Kan inte se någon gata från mitt sovrumsfönster

Fråga 11.

a) Har Du tillgång till tysta rum i Din bostad, där Du inte märker buller från väg-, tåg- eller flygtrafik?

- ☐ Ja (Gå till Fråga 11 b))
- ☐ Nej (Gå till Fråga 12)

b) Vilket/vilka rum? Flera alternativ är möjliga.

- ☐ Vardagsrum
- ☐ Sovrum
- ☐ Kök
- ☐ Annat rum

Fråga 12. Vilken typ av fönster har Din bostad?

- ☐ 2-glasfönster
- ☐ 3-glasfönster
- ☐ Annat, vad? ____________________
- ☐ Vet ej

Fråga 13. Förekommer det kondens (imma/fukt) på insidan av fönsterrutorna vintertid i sovrum eller vardagsrum?

- ☐ Ja
- ☐ Nej

Fråga 14. Har eller har Du haft synlig fuktskada (fläckar och dylikt) i Din bostad?

- ☐ Ja
- ☐ Nej

Fråga 15. Har Du under de senaste 12 månaderna känt lukt av mögel i Din bostad?

- ☐ Ja
- ☐ Nej

Fråga 16. Har Du under de senaste 12 månaderna haft synlig mögelväxt i Din bostad?

- ☐ Ja
- ☐ Nej

Fråga 17. Hur ventileras Din bostad?

- ☐ Endast självdrag
- ☐ Självdrag, samt köksfläkt och/eller badrumsfläkt
- ☐ Fläktsystem med mekanisk frånluft eller "förstärkt självdrag"
- ☐ Fläktsystem med mekanisk från- och tilluft
- ☐ Annat, vad? ______________________________
- ☐ Känner inte till ventilationstypen

Fråga 18. Sover Du med öppet fönster?

- ☐ Ja, alltid
- ☐ Ja, sommartid
- ☐ Ja, ibland
- ☐ Nej, aldrig

Fråga 19. Hur värms Din bostad i huvudsak upp? Flera alternativ är möjliga.

- ☐ Fjärrvärme
- ☐ Direktverkande el
- ☐ Elpanna
- ☐ Oljeeldning
- ☐ Eldning i panna med ackumulatortank (ved, flis, pellets etc)
- ☐ Eldning i panna utan ackumulatortank (ved, flis, pellets etc)
- ☐ Värmekamin (braskamin, kakelugn, vedspis etc)
- ☐ Annan eldning (gas, kol etc)
- ☐ Känner inte till hur bostaden värms upp

Fråga 20. Eldar Du någonsin i annat syfte än uppvärmning av Din bostad? Besvara varje rad.

	Ja, varje dag	Ja, varje vecka	Ja, men mer sällan	Nej, aldrig
Matlagning på gasspis	☐	☐	☐	☐
Matlagning på vedspis	☐	☐	☐	☐
Trivseleldning (öppen spis, braskamin, kakelugn etc)	☐	☐	☐	☐
Grillning utomhus (sommarhalvåret, ej elektrisk grill)	☐	☐	☐	☐
Eldning av löv och kvistar utomhus	☐	☐	☐	☐

Fråga 21. Eldar någon granne, som bor mindre än 50 meter från Dig, regelbundet med ved under vinterhalvåret?

☐ Ja

☐ Nej

☐ Vet ej

Utomhusmiljö

Fråga 22. Har Du tillgång till någon tyst plats utomhus i anslutning till Din bostad där Du inte märker buller från väg-, tåg- eller flygtrafik?

☐ Ja

☐ Nej

Fråga 23. I ett bostadsområde kan det förekomma olägenheter av olika slag; ett antal av de vanligaste finns uppräknade nedan. Om Du tänker på förhållandena så som Du upplevt dem de senaste 12 månaderna, hur störd eller besvärad är Du när Du befinner Dig i Din bostad?

	Märker inte	Märker, men störs inte	Störs inte särskilt mycket	Störs ganska mycket	Störs mycket	Störs oerhört mycket
Totalt trafikljud (tåg, väg, flyg)	☐	☐	☐	☐	☐	☐
Industribuller	☐	☐	☐	☐	☐	☐
Gatuliv och nattliv (restauranger, disko etc)	☐	☐	☐	☐	☐	☐
Ljud från grannar	☐	☐	☐	☐	☐	☐
Ljud från ventilation /fläktar	☐	☐	☐	☐	☐	☐
Ljud från installationer (hiss, avlopp/vatten, tvättstuga etc)	☐	☐	☐	☐	☐	☐
Lukt från industrier /verksamheter	☐	☐	☐	☐	☐	☐
Föroreningar från vedeldning	☐	☐	☐	☐	☐	☐
Avgaser från vägtrafik	☐	☐	☐	☐	☐	☐
Vibrationer från trafik	☐	☐	☐	☐	☐	☐
Annat, vad? ________ ________	☐	☐	☐	☐	☐	☐

Fråga 24. Hur mycket vistas Du utomhus i trafik i genomsnitt varje dag (t ex bil, buss, cykling, promenad på gator etc)?

- [ ] 0 - 30 minuter
- [ ] 30 minuter - 1 timme
- [ ] 1 - 2 timmar
- [ ] Mer än 2 timmar

Fråga 25. Hur mycket vistas Du utomhus annat än i trafik i genomsnitt varje dag (t ex i natur, i trädgården, på landet etc)?

- [ ] 0 - 30 minuter
- [ ] 30 minuter - 1 timme
- [ ] 1 - 2 timmar
- [ ] Mer än 2 timmar

Arbete

Fråga 26. Var vistas Du huvudsakligen dagtid? Tänk på de senaste 12 månaderna.

- [ ] På arbetsplatsen
- [ ] I skolan
- [ ] I hemmet
- [ ] På annan plats

Om Du är anställd eller egen företagare, fortsätt med fråga 27.
Om Du går i skolan, forsätt med fråga 32.
I annat fall fortsätt med fråga 36.

Fråga 27. Vilket är Ditt nuvarande yrke och/eller huvudsakliga arbetsuppgifter?

☐☐☐☐☐☐☐☐☐☐☐☐☐☐☐☐☐☐☐☐

Yrke

Arbetsuppgifter

Fråga 28. När blev Du anställd på Din nuvarande arbetsplats?

☐☐☐☐ - ☐☐

år mån

Fråga 29. Vilken är Din huvudsakliga arbetstid?

☐ Dag

☐ Natt

☐ Skift

☐ Annan

Fråga 30. Förekommer det på Din arbetsplats besvärande exponering för:

	Ja	Nej
damm?	☐	☐
kemiska ämnen, ångor/gaser?	☐	☐
motoravgaser?	☐	☐

Fråga 31. Är bullernivåerna på Din arbetsplats så höga att man rekommenderas använda hörselskydd?

- [ ] Ja
- [ ] Nej

Fråga 32. Var är Din huvudsakliga arbetsplats/skola lokaliserad? Om arbetsplatsen varierar, ange kontor eller motsvarande varifrån arbetet utgår.

____________________	____________________
Företagets/skolans namn	Avdelning
____________________	____________________
Gatunamn	Ort

__________ (Bottenvåningen = våning 0, en trappa upp = våning 1, osv.)

Våning

Fråga 33. Hur tar Du Dig vanligtvis till arbetet/skolan? Flera alternativ är möjliga.

- [ ] Bil
- [ ] Buss
- [ ] Tåg
- [ ] Cykel
- [ ] Promenad
- [ ] Annat, vad? ____________________

Fråga 34. Hur lång tid tar det för Dig att ta dig till arbetet/skolan (enkel resa)?

- ☐ Mindre än 15 minuter
- ☐ 15 - 30 minuter
- ☐ 30 minuter - 1 timme
- ☐ 1 - 1 ½ timme
- ☐ 1 ½ - 2 timmar
- ☐ Mer än 2 timmar

Fråga 35. Vilken trafikintensitet är det på gatan utanför Din arbetsplats/skola (inom ett avstånd på 50 meter)?

- ☐ 0 - 1 fordon/minut
- ☐ 2 - 5 fordon/minut
- ☐ 6 - 10 fordon/minut
- ☐ Fler än 10 fordon/minut
- ☐ Arbetsplatsen varierar

Fråga 36. Vilka sysselsättningar har Du haft sedan Du slutade skolan (ej nuvarande sysselsättning)? Ta även med arbete hemma (hemmafru, föräldraledighet etc), studier och längre perioder av arbetslöshet.

Börja med den första sysselsättningen sedan Du slutat skolan och gå framåt i tiden. Ange endast perioder som varat i mer än 12 månader.

1 ☐☐☐☐ - ☐☐☐☐
Mellan åren

Arbetsuppgift/sysselsättning

Företagets namn, Ort

Exponering för kemiska ämnen ☐ Ja ☐ Nej Exponering för buller ☐ Ja ☐ Nej

---

2 ☐☐☐☐ - ☐☐☐☐
Mellan åren

Arbetsuppgift/sysselsättning

Företagets namn, Ort

Exponering för kemiska ämnen ☐ Ja ☐ Nej Exponering för buller ☐ Ja ☐ Nej

---

3 ☐☐☐☐ - ☐☐☐☐
Mellan åren

Arbetsuppgift/sysselsättning

Företagets namn, Ort

Exponering för kemiska ämnen ☐ Ja ☐ Nej Exponering för buller ☐ Ja ☐ Nej

---

4 ☐☐☐☐ - ☐☐☐☐
Mellan åren

Arbetsuppgift/sysselsättning

Företagets namn, Ort

Exponering för kemiska ämnen ☐ Ja ☐ Nej Exponering för buller ☐ Ja ☐ Nej

---

5 ☐☐☐☐ - ☐☐☐☐
Mellan åren

Arbetsuppgift/sysselsättning

Företagets namn, Ort

Exponering för kemiska ämnen ☐ Ja ☐ Nej Exponering för buller ☐ Ja ☐ Nej

---

Om Du haft fler sysselsättningar än vad som får plats på denna sida, var vänlig skriv dem på baksidan av enkäten.

Hälsa

Fråga 37. Har eller har Du haft någon av följande sjukdomar? Besvara varje rad.

	Ja	Nej
Astma	☐	☐
Allergiska ögonbesvär	☐	☐
Hösnuva eller annan allergisk snuva	☐	☐
Andra besvär från näsan (ofta återkommande nysning, klåda, nästäppa, etc)	☐	☐
Kronisk luftrörskatarr (bronkit) eller emfysem	☐	☐
Allergiskt eksem	☐	☐

Fråga 38.

a) Har Du av en läkare fått diagnosen kronisk luftrörskatarr (bronkit) eller emfysem?

☐ Ja (Gå till Fråga 38 b))

☐ Nej (Gå till Fråga 39)

b) Hur gammal var Du när Du fick diagnosen kronisk luftrörskatarr (bronkit) eller emfysem?

☐☐ år

Fråga 39.

a) Brukar Du hosta, dagtid eller nattetid de flesta dagar under året, under så mycket som 3 månader per år?

☐ Ja (Gå till Fråga 39 b))

☐ Nej (Gå till Fråga 40)

b) Under hur många år har Du haft sådan hosta?

☐☐ år

Fråga 40.

a) Brukar Du hosta upp eller harkla upp slem från bröstet eller har Du slem i bröstet som Du har svårt att få upp, dagtid eller nattetid de flesta dagar, under året, under så mycket som 3 månader per år?

☐ Ja (Gå till Fråga 40 b))

☐ Nej (Gå till Fråga 41)

b) Under hur många år har Du haft slem i bröstet?

☐☐ år

Fråga 41.

a) Har du av läkare fått diagnosen astma?

☐ Ja (Gå till Fråga 41 b))

☐ Nej (Gå till Fråga 42)

b) Hur gammal var Du när Du fick astma?

☐☐ år

Fråga 42. Har Du under de senaste 12 månaderna haft astmabesvär? Med astmabesvär menas periodvisa eller anfallsvisa andningsbesvär eller andfåddhet.

☐ Ja

☐ Nej

Fråga 43. Använder Du någon medicin mot astma?

☐ Ja, regelbundet

☐ Ja, men endast vid behov

☐ Nej

Fråga 44.

a) Har Du av läkare fått diagnosen högt blodtryck?

☐ Ja (Gå till Fråga 44 b))

☐ Nej (Gå till Fråga 45)

b) Hur gammal var Du när Du fick diagnosen högt blodtryck?

☐☐ år

Fråga 45. Använder Du någon medicin mot högt blodtryck?

☐ Ja

☐ Nej

Vi människor är olika till vår läggning och personlighet. Vi är också mer eller mindre känsliga för störande faktorer i vår omgivning.

Fråga 46.

a) Ta ställning till vilket svar som närmast motsvarar Ditt vanliga sätt att känna när Du utsätts för:

	Inte alls känslig	Inte särskilt känslig	Ganska känslig	Mycket känslig
Buller/ljud	☐	☐	☐	☐
Damm/föroreningar	☐	☐	☐	☐
Andra störande faktorer	☐	☐	☐	☐

b) Hur karakteriserar Du Dig själv?

	Stämmer inte alls	Stämmer inte särskilt bra	Stämmer ganska bra	Stämmer precis
Jag har ganska dåligt självförtroende.	☐	☐	☐	☐
Jag hör nog till den sortens människor som är överdrivet känsliga och tar åt mig för det minsta.	☐	☐	☐	☐
Jag vågar sällan yttra mig i en diskussion, därför att jag tror att andra tycker mina åsikter inte är värda någonting.	☐	☐	☐	☐
Det tar nog ovanligt lång tid för mig att komma över obehagliga upplevelser.	☐	☐	☐	☐
Jag känner mig ofta osäker när jag träffar folk som jag inte känner så väl.	☐	☐	☐	☐
Jag brukar ofta gå och oroa mig även för sådant som andra uppfattar som bagateller.	☐	☐	☐	☐
Jag blir ängslig långt i förväg när jag ska sätta igång med något.	☐	☐	☐	☐

Övrigt

Fråga 47. Vad skulle Du själv vilja att man satsade på när det gäller miljön? Markera de tre viktigaste alternativen.

- ☐ Mer biogas och vindkraft
- ☐ Minskade industriutsläpp
- ☐ Minskade utsläpp från jordbruket
- ☐ Minskad spridning av giftiga ämnen
- ☐ Minskade utsläpp av växthusgaser
- ☐ Skydda hotade djur och växter
- ☐ Minskade utsläpp från trafiken
- ☐ Minskat buller
- ☐ Bättre skydd för dricksvattnet
- ☐ Fler naturområden
- ☐ Annat, vad?
- ☐ Inget, det behövs inte

TACK FÖR DIN MEDVERKAN!
