# Supplementary material for: Adult asthma and traffic exposure at residential address, workplace address, and self-reported daily time outdoor in traffic: A two-stage case-control study
Source: BMC Public Health. 2010 Nov 22;10:716. doi: 10.1186/1471-2458-10-716 (PMC3003254; doi:10.1186/1471-2458-10-716)
Supplement: Additional file 4 — Survey2_2005_English translation. English translation of the second survey questionnaire (2005). [file 1471-2458-10-716-S4.PDF]

Code no. (for use by YMK (Clinic of Occupational and Environmental Medicine))

Background data

Question 1. What year were you born?

1	9		

Question 2. Are you a man or a woman?

Woman

Man

Question 3. On what date did you fill out the questionnaire?

- -

year month day

Conditions growing up

Question 4. Where have you lived earlier in your life (i.e. not your current place of residence)? You can tick more than one alternative in each row.

Your age	Big city (e.g. Stockholm, Göteborg, Malmö)	Small city (e.g. Lund, Kristianstad, Helsingborg, Trelleborg)	Populated area (e.g. Hässleholm, Ystad, Simrishamn, Höör)	Village (settlement with less than 200 inhabitants)
0 - 6				
7 - 16				
17 - 30				
31 - 65				

Current residence

Question 5. How long have you lived in your current residence?

years

Question 6. How many stories does the house in which you live have?

stories

Question 7. What floor do you live on? Applies only if you live in a multi-family dwelling. (Bottom floor = floor 0, one flight up = floor 1 etc.)

Floor

Question 8. When was the house you live in built?

Before 1941

1941 - 1960

1961 - 1975

1976 - 1985

After 1985

Don’t know

Question 9.

a) Does your residence have windows facing (within a distance of 50 metres):

- ☐ a street?
- ☐ train tracks?
- ☐ an industrial area?
- ☐ a courtyard, garden, water or green area?
- ☐ Something else, what? ____

b) What is the traffic intensity on the heaviest road you can see from any window in your residence (within a distance of 50 metres)?

- ☐ 0 - 1 vehicle/minute
- ☐ 2 - 5 vehicles/minute
- ☐ 6 - 10 vehicles/minute
- ☐ More than 10 vehicles/minute
- ☐ Cannot see any street within 50 metres

Question 10.

a) Does your bedroom have a window facing (within a distance of 50 metres):

- ☐ a street?
- ☐ train tracks?
- ☐ an industrial area?
- ☐ a courtyard, garden, water or green area?
- ☐ Something else, what? ____

b) What is the traffic intensity on the heaviest road you can see from your bedroom window (within a distance of 50 metres)?

0 - 1 vehicle/minute

2 - 5 vehicles/minute

6 - 10 vehicles/minute

More than 10 vehicles/minute

Cannot see any street from my bedroom window

Question 11.

a) Do you have access to a quiet room in your residence, one where you don't notice noise from street, train or air traffic?

Yes (Go to Question 11 b)

No (Go to Question 12)

b) Which room(s)? You can tick more than one alternative.

Living room

Bedroom

Kitchen

Another room

Question 12. What types of windows does your residence have?

double-paned windows

triple-paned windows

Other, what? ______

Don’t know

Question 13. During the winter, does condensation (mist/moisture) form on the insides of your bedroom or living room windows?

Yes

No

Question 14. Have you ever had visible moisture damage (spots and the like) in your residence?

Yes

No

Question 15. Have you smelled the odour of mould in your residence in the last 12 months?

Yes

No

Question 16. Have you had visible mould growth in your residence in the last 12 months?

Yes

No

Question 17. How is your residence ventilated?

Natural ventilation only

Natural ventilation plus kitchen fan and/or bathroom fan

Fan system with mechanical exhaust air or “enhanced natural ventilation"

Fan system with mechanical exhaust and supply air

Other, what? ______

Don’t know type of ventilation

Question 18. Do you sleep with a window open?

Yes, always

Yes, in summertime

Yes, sometimes

No, never

Question 19. How is your residence heated mostly? You can tick more than one alternative.

District heating

Direct electric heating

Electric heating furnace

Oil heating

Boiler with accumulator tank (burns wood, chips, pellets etc.)

Boiler with no accumulator tank (burns wood, chips, pellets etc.)

Heating radiator (fireplace, tiled stove, woodstove etc.)

Other heating (gas, coal etc.)

Don’t know how the residence is heated

Question 20. Do you burn fuel for any purpose other than to heat your residence? Tick each row.

	Yes, everyday	Yes, every week	Yes, but less often	No, never
Cooking on gas stove				
Cooking on wood stove				
For cosiness (open pit, fireplace, tiled stove etc.)				
Grilling outdoors (summer half of year, not electric grill)				
Outdoor burning of leaves and branches				

Question 21. Does any neighbour living within 50 metres of you regularly burn wood during the winter half of the year?

Yes

No

Don’t know

Outdoor environment

Question 22. Do you have access to any outdoor quiet place associated with your residence where you don’t notice noise from road, train or air traffic?

Yes

No

Question 23. In a residential area there might be a number of annoying factors. Some of the more common are listed below. If you reflect on the conditions as you have perceived them during the last 12 months, to what degree would you consider yourself annoyed when you are in your home?

	Not aware	Aware, but it does not annoy me	Not especially annoyed	Fairly annoyed	Very annoyed	Extremely annoyed
Total traffic noise (train, road, air)						
Industrial noise						
Street and night life (restaurants, discos etc.)						
Sound from neighbours						
Sound from ventilation systems						
Sounds from other installations (e.g., elevators, drainage, laundry room etc.)						
Smells from industrial production						
Pollution from wood burning						
Exhaust fumes from road traffic						
Vibrations from traffic						
Other, what? ___						

Question 24. On average, how much time do you spend outdoors in traffic everyday (e.g. cars, buses, cycling, walking on the street etc.)?

0 - 30 minutes

30 minutes - 1 hour

1 - 2 hours

More than 2 hours

Question 25. On average, how much time do you spend outdoors other than in traffic (e.g. in nature, in the garden, in the country etc.)?

0 - 30 minutes

30 minutes - 1 hour

1 - 2 hours

More than 2 hours

Work

Question 26. Where do you spend most of your daytime? Think about the last 12 months.

At work

In school

At home

Elsewhere

If you are employed or a private business owner, proceed to Question 27. If you are going to school, proceed to Question 32. Otherwise proceed to Question 36.

Question 27. What is your current occupation and/or primary job duties?

Occupation

Job duties

Question 28. When were you hired at your current workplace?

-

year month

Question 29. What are your main work hours?

Days

Nights

Shift work

Other

Question 30. At your workplace, are you exposed to the following:

	Yes	No
Dust?		
Chemical substances, vapours/gases?		
Engine exhaust?		

Question 31. Are the noise levels at your workplace high enough that hearing protection is recommended?

Yes

No

Question 32. Where is your main workplace/school located? If your workplace varies, indicate the office or the equivalent from which the work comes.

Name of company/school

Department

Street name

Place

_______(Bottom floor = floor 0, one flight up = floor 1 etc.)

Floor

Question 33. How do you usually get to work/school? You can tick more than one alternative.

Car

Bus

Train

Bicycle

Walk

Other, what? ______

Question 34. How long does it take for you to get to work/school (one way)?

Less than 15 minutes

15 - 30 minutes

30 minutes - 1 hour

1 - 1 ½ hours

1 ½ - 2 hours

More than 2 hours

Question 35. What is the traffic intensity on the street outside of your workplace/school (within a distance of 50 metres)?

0 - 1 vehicle/minute

2 - 5 vehicles/minute

6 - 10 vehicles/minute

More than 10 vehicles/minute

My workplace varies

Question 36. How have you spent your time since leaving school (not including your current occupation)? Include work at home (housewife, parental leave etc.), studies and longer periods of unemployment.

Start with the first thing you did since leaving school and move forward in time. Include only periods that lasted more than 12 months.

1 -

Between the years

Job duty/occupation

Name of company, location

Exposure to chemical substances Yes No Exposure to noise Yes No

2 -

Between the years

Job duties/occupation

Name of company, location

Exposure to chemical substances Yes No Exposure to noise Yes No

3 -

Between the years

Job duties/occupation

Name of company, location

Exposure to chemical substances Yes No Exposure to noise Yes No

4 -

Between the years

Job duties/occupation

Name of company, location

Exposure to chemical substances Yes No Exposure to noise Yes No

5 - the years

Between

Job duties/occupation

Name of company, location

Exposure to chemical substances	Yes	No	Exposure to noise	Yes	No

If you have had more occupations than there is room for on this page, please list them on the back of the questionnaire.

Health

Question 37. Do you have or have you had any of the following illnesses? Tick every row.

	Yes	No
Asthma		
Allergic eye symptoms		
Hay fever or other allergic nasal symptoms		
Other nasal symptoms (frequent sneezing, itching, nasal drip etc.)		
Chronic bronchitis or emphysema		
Allergic eczema		

Question 38.

a) Have you been diagnosed with chronic bronchitis or emphysema by a doctor?

Yes (Go to Question 38 b))

No (Go to Question 39)

b) How old were you when you were diagnosed with chronic bronchitis or emphysema?

years

Question 39.

a) In a given year, do you tend to cough during the day or night on most days for as much as three months per year?

Yes (Go to Question 39 b))

No (Go to Question 40)

b) How many years have you had such a cough?

years

Question 40.

a) In a given year, do you tend to cough up or hack up phlegm from your chest, or do you have phlegm in your chest that you have difficultly bringing up during the day or night on most days for as much as three months out of the year?

Yes (Go to Question 40 b))

No (Go to Question 41)

b) How many years have you had phlegm in your chest?

years

Question 41.

a) Have you been diagnosed with asthma by a doctor?

Yes (Go to Question 41 b))

No (Go to Question 42)

b) How old were you when you developed asthma?

years

Question 42. Have you had asthma symptoms in the last 12 months? “Asthma symptoms” refers to periodic episodes or attacks of difficulty breathing or shortness of breath.

Yes

No

Question 43. Do you use any medication for asthma?

Yes, regularly

Yes, but only as needed

No

Question 44.

a) Have you been diagnosed with high blood pressure by a doctor?

Yes (Go to Question 44 b))

No (Go to Question 45)

b) How old were you when you were diagnosed with high blood pressure?

years

Question 45. Do you use any medication for high blood pressure?

Yes

No

We humans differ in terms of our dispositions and personalities. We are also more or less sensitive to bothersome factors in our surroundings.

Question 46.

a) Choose the answer that best corresponds to how you usually feel when you are exposed to:

	Not at all sen-sitive	Not espe-cially sensitive	Fairly sen-s-itive	Very sen-s-itive
Noise/sounds				
Dust/pollutants				
Other bothersome factors				

b) How would you characterize yourself?

	Does not ap-ply at all	Does not apply partic-ularly well	Applies fairly	Applies com-pletely
I have a rather poor self-confidence.				
I'm the kind of person who is ex-cessively sensitive and easily hurt.				
I seldom dare to speak up when in a discussion; because I believe that other people do not think my opinion matter.				
I think it takes an unusually long time for me to get over distress-ing experiences.				
I often feel insecure when I meet people that I don't know that well.				
I often worry over things that other people consider as insignificant.				
I get worried far in advance when I'm about to get started with something.				

Other

Question 47. Where do you personally feel we should concentrate our efforts when it comes to the environment? Tick the three most important alternatives.

More biogas and wind power

Reduce industrial emissions

Reduce emissions from agriculture

Reduce spread of toxic substances

Reduce greenhouse gas emissions

Protect threatened plants and animals

Reduce emissions from transportation

Reduce noise

Better protection for drinking water

More natural areas

Other, what?

Nothing, it's not necessary

THANKS FOR YOUR COOPERATION!
